# Beta-2 adrenergic receptor agonism alters astrocyte phagocytic activity and has potential applications to psychiatric disease

**DOI:** 10.1007/s44192-023-00050-5

**Published:** 2023-11-30

**Authors:** Ellen R. Bowen, Phillip DiGiacomo, Hannah P. Fraser, Kevin Guttenplan, Benjamin A. H. Smith, Marlene L. Heberling, Laura Vidano, Nigam Shah, Mehrdad Shamloo, Jennifer L. Wilson, Kevin V. Grimes

**Affiliations:** 1 Department of Chemical and Systems Biology, Stanford University School of Medicine, Stanford, CA USA; 2 Weill Cornell Medicine, New York, NY USA; 3 University of Michigan Medical School, Ann Arbor, MI USA; 4 Vollum Institute, Oregon Health & Science University, Portland, OR USA; 5 Department of Neurosurgery, Stanford University School of Medicine, Stanford, CA USA; 6 Center for Biomedical Informatics Research, Stanford School of Medicine, Stanford, CA USA; 7 Department of Bioengineering, University of California Los Angeles, Los Angeles, CA USA

## Abstract

Schizophrenia is a debilitating condition necessitating more efficacious therapies. Previous studies suggested that schizophrenia development is associated with aberrant synaptic pruning by glial cells. We pursued an interdisciplinary approach to understand whether therapeutic reduction in glial cell—specifically astrocytic—phagocytosis might benefit neuropsychiatric patients. We discovered that beta-2 adrenergic receptor (ADRB2) agonists reduced phagocytosis using a high-throughput, phenotypic screen of over 3200 compounds in primary human fetal astrocytes. We used protein interaction pathways analysis to associate ADRB2, to schizophrenia and endocytosis. We demonstrated that patients with a pediatric exposure to salmeterol, an ADRB2 agonist, had reduced in-patient psychiatry visits using a novel observational study in the electronic health record. We used a mouse model of inflammatory neurodegenerative disease and measured changes in proteins associated with endocytosis and vesicle-mediated transport after ADRB2 agonism. These results provide substantial rationale for clinical consideration of ADRB2 agonists as possible therapies for patients with schizophrenia.

**Supplementary Information:**

The online version contains supplementary material available at 10.1007/s44192-023-00050-5.

## Introduction

Afflicting nearly 1% of the world’s population, schizophrenia (SZ) is a debilitating chronic psychiatric disorder characterized by recurrent episodes of psychosis [1]. Because of its high prevalence, early onset (typically during young adulthood), and debilitating occupational and social effects, the World Health Organization ranks SZ as one of the top ten illnesses contributing to the global burden of disease. Individuals with SZ often have both positive and negative symptoms. Positive symptoms include delusions, hallucinations, disorganized behavior, and impairments in executive function; negative symptoms include flat affect, lack of energy, and social withdrawal (APA DSM-V). Diagnosis of SZ is clinical, based upon the presence of these symptoms. Currently, neither biomarkers nor physical exam findings aid in the diagnosis of SZ. Treatment of SZ patients usually begins with antipsychotic medications, such as haloperidol and risperidone that antagonize the brain’s dopaminergic and serotonergic systems. Although these medications reduce positive symptoms of SZ in 70% of patients, their side effects may cause additional hardship and often undermine patient compliance [2, 3]. Furthermore, no antipsychotic medications have shown efficacy in treating negative symptoms of the disease, and there are no currently available disease-modifying therapeutic options [4]. The combined healthcare-related and indirect costs of SZ were estimated to reach $63 billion in 2002 in the U.S. alone [5].

A principal pathological finding in postmortem studies of SZ patient brains is cortical thinning and reduced dendritic spine density on cortical pyramidal neurons [6–8]. Additionally, a recent positron emission tomography (PET) imaging study utilizing a probe for the synaptic vesicle protein SV2A, located exclusively at presynaptic terminals, found that patients with schizophrenia exhibit decreased brain synaptic density compared to healthy individuals [9]. These reductions in synapse density could result from excessive glial cell phagocytosis and have led to the hypothesis that dysregulated synaptic pruning may underlie schizophrenia pathogenesis. Dysregulation of synaptic pruning may manifest as poor connectivity between neurons and decreased excitability. This is further supported by studies showing that blocking N-methyl-D-aspartate (NMDA) receptors, the main mediators of neuronal excitation, precipitates positive SZ-like symptoms in humans [10].

Given these discoveries, we hypothesized that modulating synaptic pruning may provide therapeutic benefit for SZ. Both in normal physiology and disease, synaptic pruning is a complex biochemical process mediated by an interdependent relationship between microglia activation, the immune complement system, and astrocyte activation. Astrocytes have both indirect and direct roles in synaptic pruning [11]. We therefore sought to identify compounds that modulate astrocyte engulfment of synapses by screening over 3200 compounds. We included a library of compounds with mechanisms of action that have already been described or used in humans to facilitate identification of drug repurposing opportunities. We identified ADRB2 agonism as our lead candidate mechanism of action for later analysis.

Using computational tools, we evaluated hits, including ADRB2 agonists, from our screen for their relationship to cellular pathways involved in schizophrenia disease biology and for potential impact on patient outcomes. We and others have utilized protein–protein interactions (PPIs) of drug targets to reveal disease mechanisms. PPI network analysis can also predict diseases for drug repurposing, medication side effects, and potential drug combination effects [12–15]. Further, advances in analysis of electronic health records (EHRs) have enabled the identification of drugs with previously unknown, but favorable effects on conditions including Alzheimer’s disease, hypertension [16], colitis [17], and others. In these examples, PPI network methods were integrated with EHR analysis to connect molecular mechanisms with real world data.

Given these advances, we undertook an interdisciplinary approach to identify potential drug candidates that could reduce aberrant synaptic pruning and vetted their clinical utility through computational analyses. We analyzed EHR data to assess the relationship between exposure to the ADRB2 agonist, salmeterol, and in-patient psychiatric visits. Finally, we investigated the effect of the ADRB2 agonist mabuterol in vivo on neuroinflammation in the 5XFAD mouse model. By focusing on approved drugs with known safety profiles, we hoped to accelerate the development of new therapies for SZ.

## Results:

### A high-throughput screen identifies phagocytosis-modifying compounds in primary human astrocytes

**Fig. 1:**
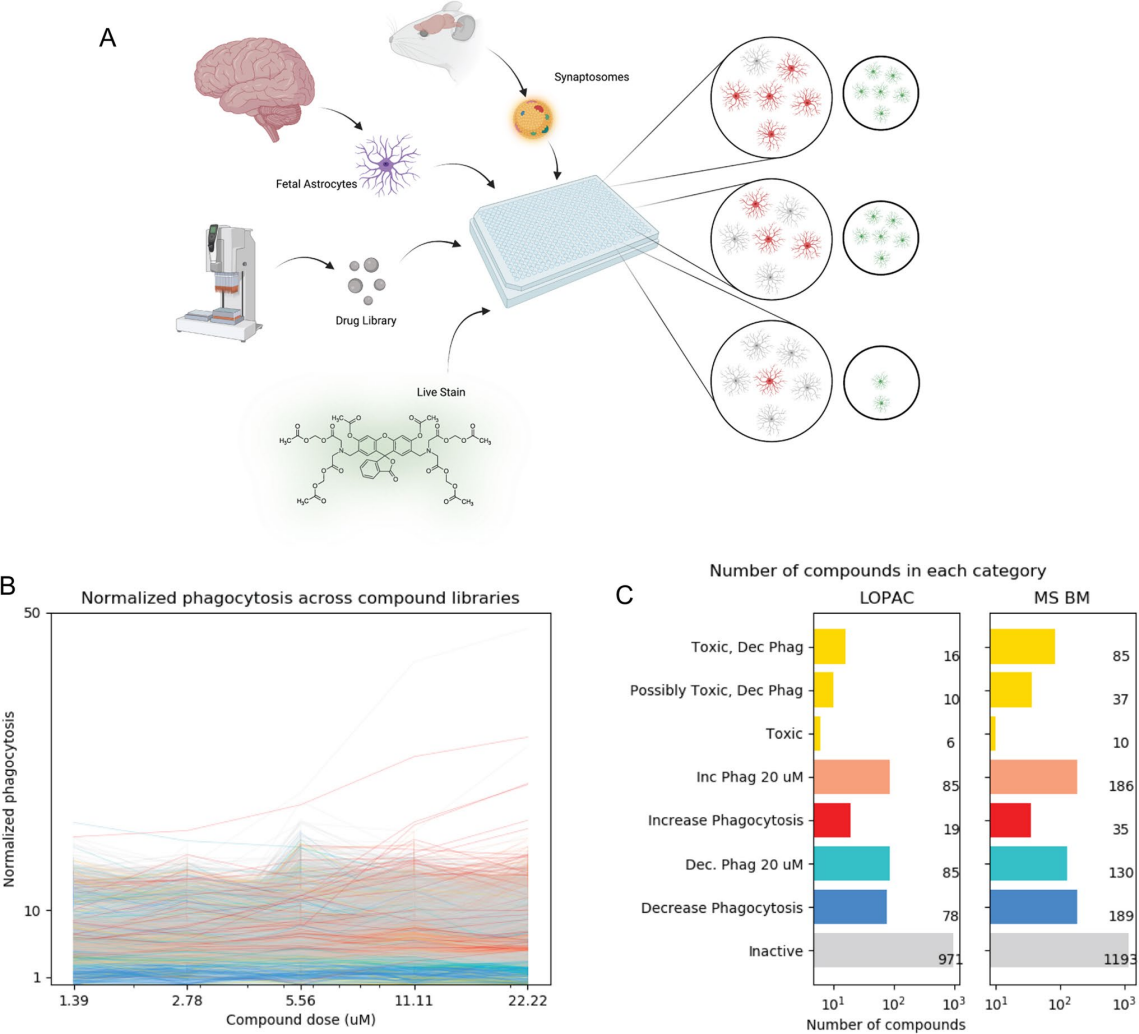
A high-throughput screening approach identified compounds that alter phagocytosis activity in primary human astrocytes. An overview of the screening approach, created with https://www.BioRender.com (**A**). Normalized dose response for all compounds in the LOPAC and MS compound libraries (**B**). All colors are consistent with **C**. Total number of compounds classified as toxic, increasing, increasing at concentrations above 20 µM, decreasing, decreasing at concentrations above 20 µM, or having no effect on phagocytosis (**C**)

To model potential impacts on synaptic pruning, we characterized compounds by their ability to affect primary human fetal astrocyte engulfment of pHrodo-labeled synaptosomes in a high-throughput, fluorescence-based screen. We adapted the published in vitro astrocyte engulfment assay [18] and followed a cell-culture protocol where astrocytes have a resting profile at baseline and accurately reflect the biology of phagocytosis found in both human and mouse mature astrocytes [19]. We measured activity in the presence of 3200 compounds from the Library of Pharmacologically Active Compounds (LOPAC) and Microsource Spectrum (MS) library (Fig. 1A). These libraries contained pharmacologically active and FDA-approved compounds, chosen to allow for more rapid drug repurposing. Briefly, we plated primary human fetal astrocytes in 384-well plates, incubated with compounds and pHrodo-labeled synaptosomes for 24 h, and imaged. Compounds were classified as decreasing or increasing phagocytosis (hits) if their engulfment of synaptosomes was < 70%, or > 130% of non-treated controls, respectively. Cells were stained with the viability dye, calcein AM, and compounds were categorized as toxic if the loss in total normalized calcein AM signal was greater than 30% of the controls. While most compounds were inactive at all tested doses, we found a small number of compounds modulated astrocyte phagocytosis (Fig. 1B, C). Importantly, we discovered that no treatments caused a noticeable change in overall cell morphology, as observed by the distribution of the viability dye within the cells, that would traditionally be associated with activation, leading us to infer that drug effects altered phagocytic biology*.* Low activation in these astrocytes is consistent with other publications using this cell culture protocol [19]*.* All screening data are included in Supplementary File 1 and additional example data for selected compounds of interest are shown in Supplementary Table 1.

**Fig. 2:**
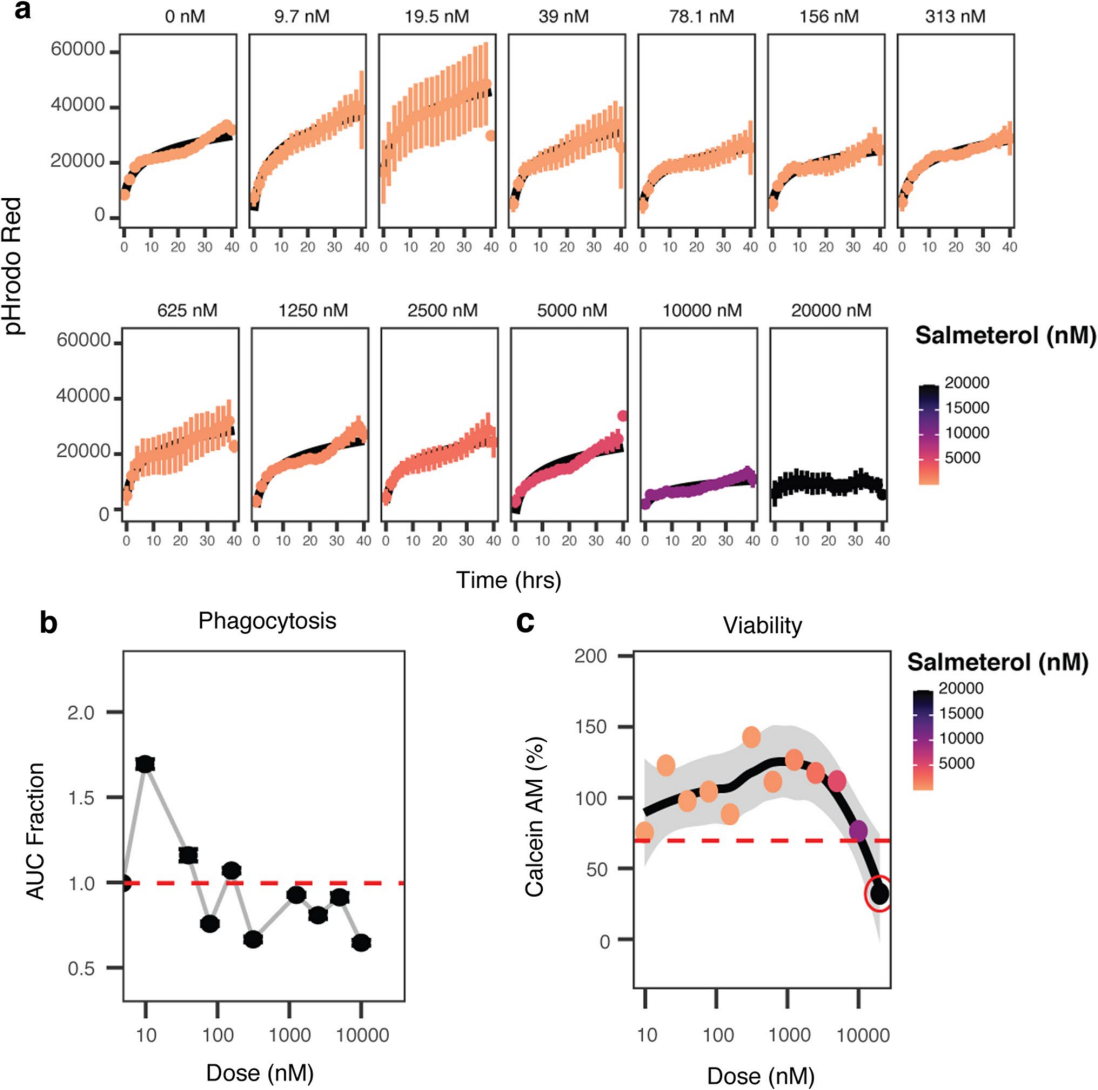
Increasing doses of salmeterol decrease phagocytosis. Engulfment of pHrodo red-labeled synaptosomes over time following salmeterol doses from 0 to 20 µM (**A**). Phagocytosis normalized to controls plotted as a function of increasing dose of salmeterol. The area under the curve (AUC) in **A** was calculated for each dose tested and normalized to the AUC of vehicle-treated controls (0 nM) (**B**). Cell viability (as measured by calcein AM dye signal) is plotted as a function of increasing dose of salmeterol (**C**). The red circle indicates a dose that was deemed toxic due to a calcein AM signal of less than 70% of vehicle-treated controls

Notably, ADRB2 agonists, such as salmeterol, decreased phagocytosis, whereas moxonidine, an alpha 2a agonist, increased phagocytosis. These observations are consistent with published examples of the opposing effects of these compounds: salmeterol stimulated cAMP production in HEK239, a kidney cell line, and in rat nervous tissue [20, 21], and stimulation of alpha 2 adrenoceptors with monoxidine caused a decrease in cAMP activity [22, 23]. This observation suggested that inhibition of phagocytosis by salmeterol was related to on-target agonism of ADRB2. In the initial screen, we investigated the effects of nine adrenoceptor agonist compounds with most being non-toxic and inactive (Supplementary Table 2). In addition to salmeterol, three of these compounds were beta-2 agonists with differing effects: salbutamol and fenoterol hydrobromide were inactive and non-toxic, and amiodarone hydrochloride decreased phagocytosis but was toxic to the cells (Supplementary Table 2). Further, salmeterol had a dose-dependent effect in the initial screen and was non-toxic at the tested doses (Supplementary Table 3). In validation assays utilizing live cell microscopy with increased replicates and expanded doses, salmeterol exhibited dose-dependent inhibition of phagocytosis (Fig. 2A, B) and was non-toxic to human astrocytes at all, but the highest doses tested (Fig. 2C). Interestingly, in validation, salmeterol increased phagocytosis at doses < 0.1 μM, however, pharmacokinetic analysis of inhaled salmeterol measured plasma concentrations ranging from 0.17 to 149 μM [24] after first inhalation or after sustained dosing, respectively, suggesting that the nanomolar effects were not relevant to in vivo response.

### Pathways analysis supports the effect of ADRB2 agonism on phagocytosis

**Fig. 3:**
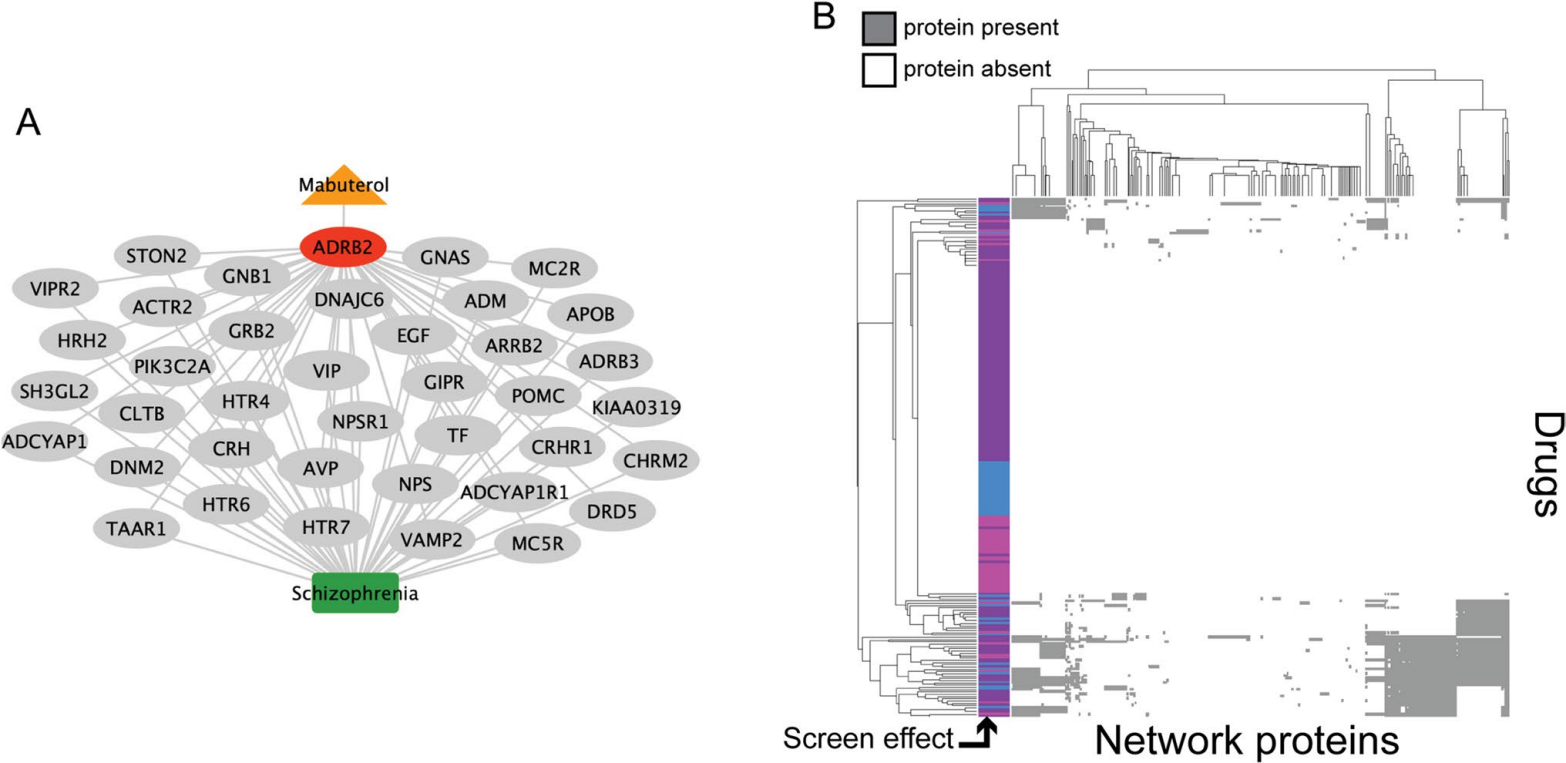
PathFX identified network associations between screen drugs and schizophrenia. An example protein–protein interaction network for mabuterol (**A**). Mabuterol, ADRB2, intermediate network proteins, and the schizophrenia phenotype are represented by an orange triangle, red ellipse, gray ellipses, and a green box, respectively. PathFX used a data-driven approach to identify proteins downstream (gray ellipses) of the ADRB2 target based on the amount and quality of evidence supporting the interactions. Edges connected to schizophrenia were selected from the full network (Supplementary File 3). We also clustered screening drugs using downstream genes discovered by PathFX (**B**). Screen drugs that increase, decrease, or had no effect on phagocytosis are shown in pink, blue, and purple, respectively, and grey or white indicates the presence or absence of a protein in a drug’s network, respectively

We conducted a pathways analysis using PathFX [15] to understand possible drug mechanisms affecting phagocytosis of synaptosomes. Briefly, PathFX used a data-driven approach, a protein-interaction network, and a gene-phenotype database to predict drug phenotypes. Importantly, PathFX was not “trained” to discover known relationships between drugs and diseases; instead PathFX used the amount and quality of evidence to predict the highest likelihood connections between drugs and biological phenotypes [15, 25, 26]. For each input drug, PathFX generated a protein–protein interaction network containing the highest quality protein interactions around a drug’s targets and an association table that quantified enriched phenotypes in the drug’s network. We first prioritized screened compounds with drug-binding proteins documented in DrugBank [27] and identified 239 compounds that spanned the decrease-, increase-, and no-effect groups from the experimental screen (drugs and PathFX results contained in Supplementary File 2). PathFX identified a network for salmeterol containing 220 proteins and 223 edges, and predicted 224 phenotypes associated with this network, including schizophrenia (4.06 × 10^–5^) and asthma (3.82 × 10^–5^) (full association table in Supplementary File 3). We next modeled mabuterol, another long-acting selective ADRB2 agonist with improved brain bioavailability compared to salmeterol and the ability to reduce memory impairment in aged mice [28]. PathFX identified a network for mabuterol containing 184 proteins and 184 edges, and predicted 302 phenotypes for this network, including Alzheimer’s disease (2.84 × 10^–5^), migraine disorders (3.10 × 10^–5^), asthma (3.47 × 10^–5^), hypertension (3.55 × 10^–5^), and schizophrenia (3.70 × 10^–5^) (full association table in Supplementary File 3). Within the mabuterol network, 38 genes were associated with schizophrenia (Fig. 3A). Interestingly, mabuterol’s drug target, ADRB2, was not directly associated to schizophrenia, but instead was associated with known schizophrenia-associated proteins through downstream protein–protein interactions (Fig. 3A). The mabuterol and salmeterol networks contained 38 and 40 schizophrenia-associated proteins, respectively. The drugs shared the 38 disease proteins discovered in mabuterol’s network because many of these were discovered through their shared target protein, ADRB2. Salmeterol’s network was slightly larger than that of mabuterol because DrugBank contained more drug-binding proteins for salmeterol and PathFX required drug-binding proteins to seed the network search.

PathFX generated connections to neuropsychiatric diseases for many of the other drugs thought to decrease phagocytosis per the astrocyte phagocytosis screen. For each drug, we investigated network proteins associated with schizophrenia. We then clustered drugs based on their PathFX network proteins (Fig. 3B**,** all cluster genes in Supplementary File 4). For many drugs, PathFX was unable to recover high confidence downstream protein interactions (white rows, Fig. 3B). This is often because a drug doesn’t have documented drug targets in DrugBank [27], or those targets have no connections in the PathFX protein–protein interaction network. Drugs with increased and decreased effects on phagocytosis clustered together because they shared similar downstream proteins. It is important to note that PathFX analysis does not distinguish if drugs differentially activate or inhibit these proteins to alter phagocytosis.

Next, we explored the extent to which screened drugs were related to neuropsychiatric diseases generally. PathFX predicted multiple phenotype associations for drugs with protein networks and we searched for an additional 77 neuropsychiatric phenotypes (Supplementary File 5). We discovered that 25/51, 21/42, and 77/136 of the increase, decrease, and no-effect on phagocytosis drugs, respectively, had at least one network association to a neuropsychiatric disease phenotype (Supplementary File 5). These results suggested that screened drugs could be broadly useful for other conditions.

We next performed gene ontology (GO) enrichment on schizophrenia-associated proteins in the mabuterol network relative to all schizophrenia genes in the PathFX database. We identified functional enrichment for processes known to be involved in schizophrenia disease biology (e.g., “dopamine receptor signaling pathway” and “associative learning”), processes related to ADRB2 agonist asthmatic therapies (e.g., “regulation of blood vessel size” and “vascular process in circulatory system”), and processes relevant to synaptic pruning (e.g., “endocytosis”) (Table 1**,** full results in Supplementary File 6). We lastly used logistic regression analysis to identify which schizophrenia-associated network genes were associated with the increase, decrease, and no-effect on phagocytosis drugs. We use regression coefficients to discover network proteins associated with each drug effect (Table 2, full results in Supplementary File 7). Compounds that increased phagocytosis were associated with ATP-Binding Cassette Subfamily B Member 1 (ABCB1; 0.727 regression coefficient). Compounds that decreased phagocytosis were associated with Glutathione S-Transferase Pi 1 (GSTP1; 0.490 regression coefficient) and Serotonin Receptor 2C (HTR2C; 0.459 regression coefficient).

**Table 1:** Abbreviated Gene Ontology (GO) enrichment of the mabuterol pathway

Description	FDR q-value
G protein-coupled receptor signaling pathway, coupled to cyclic nucleotide second messenger	8.71E−12
Adenylate cyclase-modulating G protein-coupled receptor signaling pathway	2.06E−09
cAMP-mediated signaling	1.93E−07
Membrane organization	4.71E−06
Signal transduction	8.68E−06
Regulation of blood vessel size	2.33E−03
Vascular process in circulatory system	2.58E−03
Endocytosis	9.06E−03
Serotonin receptor signaling pathway	3.47E−02
Neuropeptide signaling pathway	4.12E−02
Regulation of glucocorticoid secretion	5.42E−02
Dopamine receptor signaling pathway	7.09E−02
Female pregnancy	1.26E−01
Associative learning	1.42E−01

Remaining GO terms are in Supplementary File 6

**Table 2:** Schizophrenia-associated network proteins with the highest logistic regression coefficients for drugs that increased, decreased, or had no effect on phagocytosis

Increase phagocytosis		Decrease phagocytosis		No Effect	
Protein names	Regression coefficients	Protein names	Regression coefficients	Protein names	Regression coefficients
ABCB1	0.727	GSTP1	0.490	SLC6A3	0.781
		HTR2C	0.459	HTR1B	0.515
				KCNH2	0.512
				HTR2A	0.411

The full set of regression coefficients are contained in Supplementary File 7

### ADRB2 activation induced protein level changes associated with endocytosis and vesicle-mediated transport in the mouse brain and had no discernable differences in behavioral assays

**Fig. 4:**
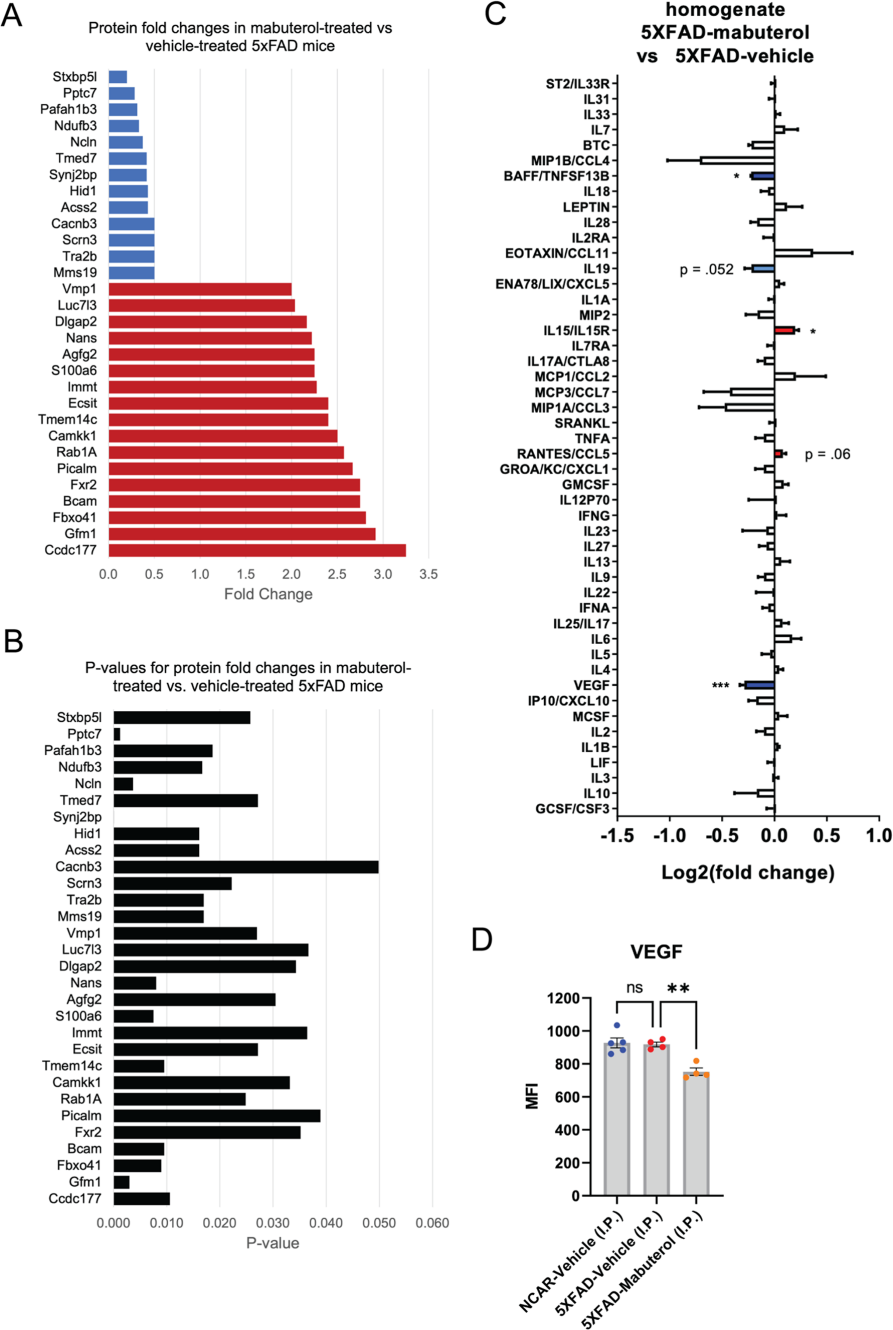
Mabuterol treatment induced changes in protein and cytokines. Protein level fold-changes from 5XFAD mouse brain tissue that were increased (red) or decreased (blue) significantly (p < 0.05) following mabuterol treatment (**A**) and corresponding p-values determined via t-test (**B**). Change in cytokine levels in brain tissue (red/blue = up/down regulated) from 5XFAD mice treated with mabuterol compared to vehicle-treated 5XFAD mice as assessed by Luminex 48-plex (Affymetrix) mouse cytokine assay (*p < 0.05, **p < 0.01, ***p < 0.001; Šidák’s posthoc after 1-way ANOVA) (**C**) Vascular endothelial factor (VEGF) levels in non-carrier (NC) and 5XFAD mice following vehicle (veh), or mabuterol (mab) treatment (**D**). MFI = median fluorescent intensity

Next, we explored how ADRB2 agonism affected molecular level changes in vivo*.* We used the 5XFAD mouse model, a neuroinflammatory model of amyloidosis. Previous work demonstrated that ADRB2 antagonism was pro-inflammatory, promoted neurodegeneration, and altered phagocytosis in 5XFAD mice [29]. However, previous work had not connected molecular level information to drug pathways or psychiatric drug repurposing. We treated 5XFAD mice with mabuterol or vehicle daily for two months and concurrently assessed behavioral activity days 25, 26, 46, and 48 of dosing. Throughout the study, all mice survived and maintained normal body weight (Supplementary Fig. 1). We monitored mice behavior via the Activity Chamber [30] and Y-maze Forced Alternation [28] tests and observed no discernable differences from baseline to 4 and 7 weeks of dosing (Supplementary Fig. 2, 3). At the end of the study, we homogenized specific brain sections and measured protein-level changes. We observed a set of 17 up- and 13 down- regulated proteins (p < 0.05) following mabuterol treatment (Fig. 4A, Supplementary Fig. 4). Because the datasets were small, we were unable to measure GO enrichment independently from only mabuterol up- or down- regulated proteins and we instead investigated the combination of up- and down- regulated proteins. While not statistically significant, GO enrichment of mabuterol-induced changes compared to all proteins in our pathways network revealed enrichment of protein complex stability regulation and cargo loading into vesicles (Table 3). Enrichment highlighted two proteins in particular – phosphatidylinositol binding clathrin assembly protein (PICALM) and rab1a, member ras oncogene family (RAB1A) – as associated with cargo loading into vessels. Rab GTPases are known to regulate phagocytic activity in the immune response [31–33] and PICALM variants are associated with poor plaque removal in Alzheimer’s patients and are hypothesized to contribute to altered phagocytosis [34].

**Table 3:** GO analysis of altered protein levels following mabuterol treatment

GO Term	Description	P-value	FDR q-value	Enrichment	N	B	n	b	Genes
GO:0061635	Regulation of protein complex stability	1.47E−04	1.00E + 00	108.24	15,154	10	28	2	ECSIT, NCLN
GO:0035459	Cargo loading into vesicle	5.52E−04	1.00E + 00	56.97	15,154	19	28	2	PICALM, RAB1A

*ECIT* ecsit signaling integrator, *NCLN* nicalin, *PICALM* phosphatidylinositol binding clathrin assembly protein, *RAB1A* rab1a, member ras oncogene family

We also observed that vacuole membrane protein 1 (VMP1) was up-regulated following mabuterol treatment (Fig. 4A). RAB1A and VMP1 are known to have a role in autophagy, suggesting altered activity in autophagic pathways. Taken together, these results provide further evidence of a connection between ADRB2 agonism and phagocytosis pathways, if not a definitive mechanism.

We also explored changes in immune cytokines via Luminex 48-plex (Affymetrix) mouse cytokine assay and observed reduction in B-cell activating factor (BAFF)/ TNF superfamily member 13b (TNFSF13B) and vascular endothelial growth factor (VEGF), and an increase in interluekin-15 and its receptor (IL15, IL15R) (Fig. 4C, Supplementary Fig. 5). The discovery of some reduced inflammatory markers in the brain following mabuterol treatment is consistent with previous work [30, 35]. Lastly, we analyzed targeted protein levels via western blot analysis and saw no significant changes following mabuterol treatment (Supplementary Fig. 6).

### Patients with a pediatric exposure to ADRB2 agonists have reduced likelihood of in-patient visits following a diagnosis of schizophrenia

We hypothesized that an observational study using the electronic health record could test whether pediatric exposure to ADRB2 agonist drugs may confer better outcomes for patients with schizophrenia. ADRB2 agonists, such as salmeterol, are routinely used to treat asthma in pediatric patients; having enough patients exposed to the drug class made it feasible to conduct an exploratory observational study. These compounds have a known safety profile and thus are attractive for drug repurposing [36]. We focused on pediatric exposures because schizophrenia is considered a developmental disease and there may be a critical developmental window when aberrant synaptic pruning occurs. Our target cohort included young adult (age > 18) patients with a diagnosis of schizophrenia who also received an ADRB2 agonist treatment (all compounds listed in Supplementary File 9) between the ages of 6–18. It was necessary to simultaneously explore all ADRB2 agonists and formulations to have sufficient patients to conduct this analysis.

**Fig. 5:**
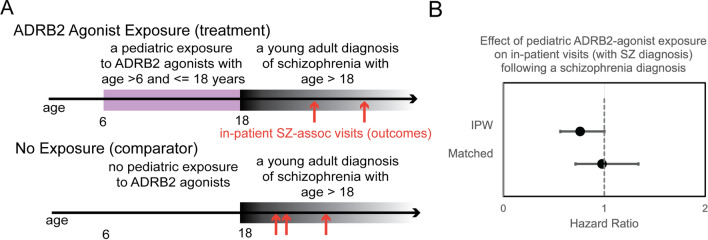
An observational study measured the effect of pediatric exposure to ADRB2 agonists on inpatient visits. The study design leveraged a target cohort that included patients with a pediatric treatment with ADRB2 agonists between the ages of 6–18 and a comparator cohort without this treatment. In both cohorts, we considered the outcome of in-patient visits that were associated with a schizophrenia diagnosis (SZ) (**A**). We measured the hazard ratio between target and comparator groups by fitting a Cox regression model (**B**). IPW represents a comparison using “inverse probability weighting” to create a balanced comparison among patients accounting for covariates, and “matched” represents a subpopulation of target-comparator patient pairs with similar covariates

For these analyses, we used the deidentified Optum Clinformatics dataset v7 that included over 88 million US patients, both privately insured and Medicare beneficiaries, largely under the age of 65. We accessed a version of the Optum dataset standardized to the Observational Medical Outcomes Partnership (OMOP) common data model (CDM); standardized data models have decreased heterogeneity between datasets and improved consistency in underlying data [37]. We implemented a Cox regression model using the CohortMethod [38] package to assess differences between the target and comparator groups in the outcome of in-patient visits associated with a schizophrenia diagnosis. We used large-scale propensity matching to control for patient confounding [39], which controlled for the fact that pediatric patients taking ADRB2 agonists, such as salmeterol, were also likely diagnosed with asthma. Using this method, we achieved reasonable covariance balance (see methods and code repository, full patient covariates table included in Supplementary File 9, Supplementary Table 4). Because our patient cohorts were small (600 and 14,904 patients in the target and comparator cohorts, respectively, Supplementary Table 5), we estimated effect sizes for inverse propensity weighted (IPW) and matched patient populations (see methods). Matched cohorts required comparisons between patient pairs with similar clinical features and narrowed the overall study population to 536 and 9,108 patients in the target and comparator cohorts, respectively (attrition diagram in Supplementary Fig. 7). IPW analysis included all patients and weighted their contribution to the effect size based on their proportional representation in the study population. In the IPW patient population, we measured a HR of 0.76 (95% CI 0.56–1.00) and in the matched population a HR of 0.97 (95% CI 0.71–1.33) (Fig. 5 and Table 4). This exploratory analysis suggests a small, albeit not statistically significant, protective effect from ADRB2 agonist exposure on outcomes associated with schizophrenia. Further studies in larger patient populations would be required to validate the effect.

**Table 4:** An observational study measured a reduced rate of inpatient hospital visits among patients with a pediatric exposure to ADRB2 agonist compounds

Patient Population	Estimate	Lower 0.95	Upper 0.95
IPW	0.76	0.56	1.00
Matched	0.97	0.71	1.33

## Discussion

Here we pursued an integrated, interdisciplinary analysis to explore compounds that altered phagocytotic activity and their potential for repurposing to treat schizophrenia. Our analysis was motivated by the hypothesis that aberrant synaptic pruning contributes to schizophrenia symptoms. We first conducted a high-throughput phenotypic screen of primary human fetal astrocytes and observed that ADRB2 agonists, specifically salmeterol, decreased phagocytosis. We conducted pathways analysis using drugs from our screen using the PathFX algorithm, which discovered associations between salmeterol’s drug target, ADRB2, and schizophrenia disease genes. Interestingly, these associations were mediated through proteins downstream of the druggable target, suggesting the importance of understanding pathways effects for drug repurposing. These pathways were enriched for genes associated with endocytosis and vesicle dynamics, further supporting a possible connection between ADRB2 agonism, schizophrenia, and altered phagocytosis. We used in vivo analysis of the drug mabuterol, a ADRB2 agonist with greater brain permeability than salmeterol, to measure protein level changes in the 5XFAD mouse. We again used GO analysis to discover that altered proteins were associated with endocytosis and autophagy, as well as neuroinflammation. Our in vivo analysis further supported a connection between ADRB2 agonism and altered phagocytic activity. However, using brain homogenate did not allow us to distinguish between cell types where protein level changes occurred, and use of adjacent brain regions limited our ability to definitively discern activation of different brain regions. Further, in this mouse model, it’s possible that phagocytosis is most active in microglial cells. While we could not directly test the role of astrocytes in this model, we hypothesized that phagocytosis pathways may be sufficiently similar for this preliminary analysis. Interestingly, analysis of altered proteins following mabuterol treatment also uncovered proteins related to autophagy, specifically RAB1A and VMP1. Finally, we completed an observational study utilizing electronic health records and demonstrated that pediatric ADRB2 agonist exposure led to a modest, but not significant, decrease in in-patient visits associated with a diagnosis of schizophrenia.

The literature supports many of the connections we made between phagocytosis and possible outcomes for patients with schizophrenia. Two independent studies of minocycline supported the role of altered phagocytosis as a mechanism of novel treatments for schizophrenia. The first study observed that patients prescribed minocycline for acne saw improvements in negative symptoms associated with schizophrenia [40] and a second study discovered that minocycline reduced depression symptoms by altering phagocytosis [41]. Literature evidence supports that proteins discovered from our in vivo analysis have roles in endocytosis, but they have not previously been described to have a role in schizophrenia. PICALM is integral to endocytosis and has been correlated with levels of autophagy-related proteins in neuropsychiatric disease contexts, especially Alzheimer's disease [42]. PICALM performs this modulation of autophagy by regulating endocytosis of soluble N-ethylmaleimide-sensitive factor attachment protein receptor (SNAREs) genes such as VAMP2 [43], a gene that was identified in the protein–protein interaction network for mabuterol generated by PathFX. Further, decreasing the expression of PICALM has been shown to reduce endocytosis and the activity of the β-secretase enzyme, thought to play a major role in the pathogenesis of Alzheimer’s disease [44]. Further, the literature supported a hypothesized mechanism through altered cAMP activity after ADRB2 agonism [20–22], however, further mechanistic studies in astrocytes would be required to verify this mechanism. Investigations using phosphodiesterase inhibitors and additional ADRB2 agonists would be required to validate this mechanism and further effects on phagocytosis.

Many other studies have emphasized the role of aberrant phagocytosis in neurodegenerative disease yet haven’t considered parallels of this process to psychiatric diseases such as schizophrenia. For instance, one study demonstrated that ADRB2 agonism attenuated and antagonism enhanced microglia phagocytosis in a non-psychiatric model of neurodegenerative disease [29]. One review highlighted that microglia and astrocytes are both active at sites of neurodegeneration, that they have shared co-activation mechanisms, and that glial cells contribute to pathological phagocytosis in neurodegeneration [45]. Schizophrenia shares genetic markers with some neurodegenerative diseases; genome-wide association studies have shown that Alzheimer’s disease and schizophrenia share genetic markers and overlapping association patterns [46]. This suggests that pathways discovered in neurodegenerative disease may have utility for advancing schizophrenia treatments.

PathFX analysis independently predicted an association between ADRB2 and schizophrenia-associated proteins. Also of note, ADRB2 was not directly associated with schizophrenia, but PathFX made this connection using proteins downstream of the receptor, suggesting that methods that search downstream of druggable targets may advance drug repurposing efforts. Pathways analysis is increasingly used to predict novel uses for existing drugs and often predicts many more drug effects than are supported by existing evidence. Another interesting ramification of our investigation is that complementary experimental work can be used to better understand which network predictions may have clinical utility. Observational studies leveraging electronic health records can further elevate evidence from experimental screens and support hypotheses generated from early preclinical data. Indeed, we used this analysis to support our overall hypothesis and saw a decrease in in-patient visits for SZ patients with a pediatric exposure to salmeterol. The observational study was underpowered to be conclusive but provides rationale for future clinical investigation.

Overall, our goal was to use an integrated approach to rapidly uncover novel treatments. Increasingly, integrated approaches are becoming standard for identifying new treatments, especially for psychiatric conditions where single systems, either computational models or animal models, are unable to identify efficacious treatments alone. Previous perspectives about the future of psychiatric drug development, including for schizophrenia, have emphasized integrated approaches, but largely emphasized the importance of including biomarkers and behavioral assays [47] and the role of hypothesis-driven drug target identification [48, 49]. We pursued a pragmatic approach that emphasized preliminary mechanistic data and observing clinical effects for the goal of expediting treatment development. Future clinical studies would be needed to validate our streamlined approach to drug repurposing and the clinical relevance of these findings.

## Methods

### Astrocytes and cell culture

Fetal human brain tissue was obtained through StemExpress under a protocol approved by the Stanford University Institutional Review Board. Astrocyte cultures were obtained and maintained according to Zhang et al., [19] (which contains a complete, step-by-step protocol). Briefly, fetal brain tissue was cut into ~ 1 mm^3^ pieces and enzymatically digested in 7.5 unit/ml papain (Worthington, LS 03126) at 34 °C for 30 min. Tissue mechanically dissociated to produce a single cell suspension in a protease inhibitor solution (Worthington LS003086). Cells were resuspended in PBS with BSA (Sigma A8806) and DNase (Worthington, LS002007) and passed it through a Nitex [(Tetko Inc, HC3-20)] filter to remove cell clumps. Cells were then incubated for 30 min at room temperature on a petri dish coated with anti-CD45 (BD Pharmingen, 550,539) antibody to negatively select for microglia/macrophages followed by a petri dish coated with anti-Thy1 (CD90; BD, 550,402) to negatively select for neurons. The cell suspension was then incubated for 45 min on an anti-HepaCAM (R&D systems, MAB4108) plate to select for astrocytes. The plate was washed with PBS to remove unattached cells and the purified astrocytes removed from the plate by 0.05% trypsin (Sigma, T9935) digestion for 3 min at 37 °C. Collected cells were stored in Bambanker reagent (BulldogBio BB01) according to manufacturer’s instructions.

Astrocytes were cultured in a defined, serum-free base medium containing 50% neurobasal (Gibco, 21,103–049), 50% DMEM (Invitrogen, 11,960–044), 100 U/ml penicillin + 100 μg/ml streptomycin (Invitrogen, 15,140–122), 1 mM sodium pyruvate (Invitrogen, 11,360–070), 292 μg/ml l-glutamine (Invitrogen, 25,030–081), 1 × SATO (see Zhang et al., [19]), 5 μg/ml of N-acetyl cysteine (NAC, Sigma, A8199), and 5 ng/ml HBEGF.

### Synaptosome preparation

Synaptosomes were prepared as published previously [18, 50]. All procedures involving animals were conducted in conformity with Stanford University guidelines that are in compliance with national and state laws and policies. Synaptosomes were prepared from whole Sprague Dawley rat cortex as published previously [50] and conjugated to pHrodo Red, succinimidyl ester (Thermo Fisher Scientific, P36600) in 0.1 M sodium carbonate (pH 9.0) at room temperature with gentle agitation [51]. After a 2-h incubation, unbounded pHrodo was washed out by multiple rounds of centrifugation and pHrodo-conjugated synaptosomes/myelin were re-suspended with isotonic buffer containing 5% DMSO for subsequent freezing.

### High-throughput screening

Astrocytes were plated on day 0 into solid-black 384 well Greiner plates PDL coated (EK-30946) plates at 1000 astrocytes/well, in 50 μL media. After 3 days in culture, we used the SciClone ALH3000 to add 200 nL of all screen compounds (Supplementary File 1) to the plates, and then added 40 μL of additional media with 0.1% serum (all columns) and 0.5 μL/well of pHrodo (pHrodo™ Red AM Intracellular pH Indicator) containing labeled synaptosomes (columns 1–22 only). After an additional 24 h incubation, we added 10 μL of 50 μM Calcein AM (Fisher C3100MP) to the plates and imaged cells (see below). The screening protocol is also included in our GitHub repository (https://github.com/jenwilson521/phagocytosis_beta2Agonists).

### Compound libraries and dosing

We screened the Library of Pharmacologically Active Compounds (LOPAC^1280^) (Sigma, St Louis, MO, USA) and Microsource Spectrum (MS) compound libraries. The LOPAC and MS libraries included 1,280 and 2000 unique compounds respectively and all compounds were dosed at 1.39, 2.78, 5.56, 11.11, and 22.22 μM in singlicate. All raw cell imaging data and plate data are included in Supplementary File 1 (sheet names “Well Data” and “Plate Data”). For each plate, we included four rows of cells with media, without any compound (“High Control”), one row of postive_control_compound (“Positive Control”) and media without cells or compound (“Low Control”).

### Image analysis and data normalization

After adding calcein AM, the plates were moved into an incubator in a separate IXMicro room where we used a CRS robot to load and image plates using an ImageXpress Micro. We used FITC-FIXED and TRITC-FIXED cubes and used a 600 ms exposure for each. Within each plate, we calculated the median total cells in the high and low controls, the median calcein AM integrated intensity (“green”) and median pHrodo phagocytosis integrated intensity (“red”) of all non-control, data wells, and calculated the median %phagocytosis integrated intensity adjusted for total calcein AM signal (a red/green ratio that represented the relative amount of phagocytosis activity while controlling for live cells). After completing plate-normalization, we calculated the total number of cells with pHrodo red and calcein green signal and calculated the red:green ratio per cell. We then further normalized this ratio to the percent of the median of red:green ratios in the plate. We considered a compound to be toxic if the integrated calcein signal within a well was 30% less than the median plate calcein signal.

Using the normalized red:green ratios, we fit a dose–response curve and estimated the EC50 of pHrodo-red induction or inhibition as a measure of the drug’s effect on phagocytosis. We considered compounds with 100%, < 70%, and > 130% of the median red:green ratio when the [concentration] > 5 μM as compounds as no-effect, decreasing, or increasing phagocytosis respectively. We generated summary images from this analysis using (count_screen_data.py) provided in the GitHub repository.

### Incucyte validation of salmeterol

1000 fetal human astrocytes per well were plated on Greiner Bio-One CELLSTAR μClear™ 96-well, Cell Culture-Treated, Flat-Bottom Microplates and cultured for 3 days to establish cell viability. Cells were then treated with various doses of salmeterol as well as 0.1% v/v of fetal bovine serum (Thermo Fisher 10,437,028) to provide opsonins for phagocytosis. One hour later, cells were treated with 5ul of pHrodo-conjugated synaptosomes and 50 μM Calcein AM (live stain) and imaged at 1 h intervals. Images were acquired with an Incucyte ZOOM (Essen Bioscience) at 37 °C and 10% CO2. For image processing analysis, we took 3 images per well using a 20 × objective lens from random areas of the 96-well plates. Custom analysis scripts within the Incucyte ZOOM Software were used to measure phagocytosed particles (pHrodo-positive area) and live cells (Calcein AM area), and intensity and particle size cutoffs were used to discriminate signal from noise.

### Running PathFX analysis

We ran PathFX analysis for 239 drugs from the three different experimental groups—increase, decrease, or no effect on phagocytosis—using the PathFX algorithm (version 1.0) [15]. We also ran PathFX using ADRB2 as a single drug target to simulate effects of ADRB2 agonists, such as mabuterol (analysis contained in the script, *run_PathFX_mabuterol.py*). We ran PathFX using default parameters and provided DrugBank identifiers as inputs. This analysis was completed in four scripts: *run_multiDrug_incease.py*, *run_multiDrug_decrease.py, run_multiDrug_noeffect.py*, and *run_PathFX_mabuterol.py*. We selected the 239 drugs from the phagocytosis screen (see above) based on whether they had protein-binding targets in DrugBank [27]. Out of the 239 total drugs selected for PathFX analysis, 52 were determined to increase phagocytosis, 43 decreased phagocytosis, and 144 had no effect on phagocytosis based on the primary in vitro screen.

### Uncovering network associations to multiple neuropsychiatric disorders

Using the network and phenotype tables identified from PathFX, we identified drug network genes associating the drug targets to neuropsychiatric disorders. We searched for 77 different psychiatric and immune phenotypes and grouped these 77 neuropsychiatric phenotypes into 10 different clusters based on clinical similarity (*Neuropsychiatric_clusters.xlsx*). We counted drug networks that were associated with these phenotypes for each of the three groups. This analysis was also completed in three scripts: *phenotype_counting.py*, *phenotype_counting_decrease.py*, and *phenotype_counting_noeffect.py*. These scripts generated an excel matrix (*Phenotype_counting.xlsx*) that stores whether a phenotype (represented by its CUI identifier) is present in the network of a drug by marking a ‘0’ (for not present) or a ‘1’ (for present) in the cells of the sheet.

### Gene counting and phenotypic grouping

We next analyzed network genes associated with psychiatric diseases. We analyzed all schizophrenia-associated genes using the gene-phenotype data from PathFX using the following script: *get_schizophrenia_genes.py*. This yielded a list of 1,947 genes, aggregated from a variety of different sources, including ClinVar, OMIM, PheGenl, and DisGeNet (as published in [15]). We identified schizophrenia-associated genes in the 239 drug pathways. This analysis was completed in three scripts: *gene_counting.py*, *gene_counting_decrease.py*, and *gene_counting_noeffect.py*.

### GO enrichment

For individual drugs, we conducted GO analysis of PathFX-identified schizophrenia-associated genes (all contained in Supplementary File 2) using the GOrilla tool (http://cbl-gorilla.cs.technion.ac.il/) [52], using a target (*mabuterol_schizophrenia_genes.txt*) and background (*all_schizophrenia_genes.txt*) list, selecting for molecular process, function, and component GO ontologies, and using Homo Sapiens as the organism. The full GO enrichment results for mabuterol are contained in Supplementary File 6. We also used GO enrichment to understand proteomic changes measured in the mouse brain. We again used the Gorilla tool but converted significantly changed mouse proteins to their human homologues and used this as the foreground list compared to all schizophrenia proteins and all network proteins. Both comparisons are contained in Supplementary File 8.

### Clustering and meta-analysis

To look for network patterns, we clustered drugs based on phenotype-associated genes across experimental groups using the *clustering.py* script using the fastcluster module in python and one-hot encoding of network proteins. We further modified our initial clustering to create a clarified heatmap image (*all_drugs_clustermap.png* for schizophrenia). This analysis is contained in *clustering_update_fig.py*.

### Logistic regression

We used logistic regression to discern network patterns associated with the ‘Increase,’ ‘Decrease,’ or ‘No effect’ phagocytosis experimental groups. For this analysis, we merged the gene counting results from each experimental group into a single matrix (*convert_to_single_matrix.py*). Then we ran the logistic regression using the LogisticRegression module in python; this analysis is contained in the following two scripts: *call_logistic_regression.py* and *run_logistic_regression.py*. These scripts generated an excel sheet for each phenotype, labeled by CUI term, which ranks the genes for each experimental group in order of regression coefficients. For example, the schizophrenia logistic regression results can be found here: *Logistic_regression_results/*G*ene_counting_C0036341_mergedregCoeff.xlsx*.

### In vivo model

We investigated the effects of a selective beta-2 adrenergic receptor (ADRB2) agonist, mabuterol in 3-month-old 5XFAD male mice from MMRRC (JAX #034840-JAX). This neuroinflammatory model of amyloidosis was selected because previous studies have evidence of altered synaptic pruning [28]. Mice were treated daily for 2 months with vehicle (0.9% Saline) or 0.3 mg/kg mabuterol. Behavioral testing, including Activity Chamber and Y-maze: Forced Alternation, was performed at set time points as described below. Methods for each behavioral test are described below. Mice were group-housed under a reversed light–dark cycle with lights off at 8:30 AM and on at 8:30 PM. Mice were handled prior to the experiments to habituate mice to interacting with the experimenter. All procedures related to animal maintenance and experimentation were approved by the Stanford University Administrative Panel for Laboratory Animal Care and conformed to the U.S. National Institutes of Health Guide for the Care and Use of Laboratory Animals. Efforts were made to minimize the number of mice used and their suffering (see Table 5).

**Table 5:** Animal cohorts for in vivo study of ADRB2 agonism

Group	Strain	Sex	Age	# of Animals	Genotype	Treatment
1	5XFAD	Male	3 months	n = 5	Non-carrier	Vehicle (0.9% Saline, I.P)
2	5XFAD	Male	3 months	n = 5	5XFAD	Vehicle (0.9% Saline, I.P)
3	5XFAD	Male	3 months	n = 5	5XFAD	Mabuterol (0.3 mg/kg, I.P)

### Activity chamber

The Activity Chamber (AC) was used to assess general locomotor activity and exploration as described previously [30]. Briefly, mice were placed in one corner of a square Open Field Activity Arena (43 × 43 × 30 cm; Med Associates Inc., St. Albans, Vermont; Model ENV-515) located inside of a dark sound-attenuated chamber (74 × 60 × 60 cm) and allowed to freely explore the arena. Movement was tracked by an automated tracking system with three planes of infrared detectors during a 10-min trial. Parameters measured included distance moved, vertical counts (rearing), and time spent in the periphery and center of the arena. The periphery was defined as the zone within 5 cm of the arena wall. Between each trial, the surface of the arena was cleaned with 1% Virkon disinfectant. AC was conducted at baseline, 4-, and 7-weeks post-dosing.

### Y-maze: forced alternation

The Y-maze was used to assess behavior believed to be associated with hippocampal-dependent spatial reference memory. This test is based on the tendency of rodents to preferentially explore a novel environment over a familiar one. In this case, a normal rodent prefers to explore a different arm of the maze than an arm they previously explored. The maze was made of plastic with 3 arms in a “Y” shape (each arm 40 × 8 × 15 cm). The test consisted of two 8-min trials separated by a 1-h intertrial interval (ITI) as described in [28]. To start each trial, mice were placed at the end of one of the arms (Start Arm). During the first trial (Training), mice were only allowed to explore two of the three arms (Familiar Arms). A plastic insert blocked off the third arm (Novel Arm). The Novel Arm was pseudorandomized to avoid any location bias. During the second trial (Testing), the insert was removed, and the mice were allowed to explore all three arms. The trials were recorded with an overhead camera and tracked with Ethovision XT (Noldus Information Technology, Wageningen, Netherlands). Between each trial, the surface of the maze was cleaned with 1% Virkon disinfectant. Y-maze was conducted 4- and 7-weeks post-dosing.

### Tissue collection

Brain and plasma were collected after 2 months of dosing. Mice were dosed 1 h (± 15 min) prior to tissue collection. Mice were deeply anesthetized with isoflurane. Prior to perfusion, whole blood was collected from the left ventricle via cardiac puncture (23 g needle) into K3EDTA-containing vials (Greiner Bio-One, MiniCollect Tube Reference #450,475). Blood was spun (10 min, 3000*g*, 4 °C) within 1 h of collection, and plasma was stored at − 80 °C. For perfusion, the right atrium was opened, and mice were transcardially perfused with ice-cold phosphate-buffered saline (PBS; pH 7.4) through a 23 g needle. The perfused brain was removed. The brain was bisected coronally at the level of the mammillary bodies into the forebrain and hindbrain. The forebrain was hemisected. The left hemisphere was immediately flash-frozen on dry ice and stored at − 80 °C for later analysis. The right hemisphere and hindbrain were post-fixed with 4% paraformaldehyde in a 15 ml conical centrifuge tube (48 h, 4 °C). Following post-fixation, the hemisphere and hindbrain were transferred to 30% sucrose in phosphate buffer (PB) and stored at 4 °C for later analysis. For subsequent analyses, we sectioned mouse tissue to accommodate the tissue and reagent requirements for each additional assay.

### Multiplex mouse cytokine assay

Multiplex tissue cytokines were analyzed in plasma and brain homogenate using a Luminex 48-plex (Affymetrix) mouse cytokine assay as described in [29]. Brain homogenate was prepared from a 2 mm coronal section of the left hemisphere just in front of the hippocampus. The Luminex assay was performed in the Human Immune Monitoring Center at Stanford University, following manufacturer instructions. Briefly, hippocampal tissue was homogenized in a protein extraction buffer (1% triton X100, 0.5% tergitol, 25 mM Tris HCl, 100 mM NaCl containing Halt protease inhibitor cocktail, 1 × and 1 uM phenylmethanesulfonyl fluoride, 1x). The tissue was lysed by pulling through a 23 g needle (10 ×) and then sonicated for 3 × 3 s pulses. Homogenate was spun at 14,000*g* for 10 min, and protein concentrations were determined by Pierce BCA assay. Homogenate samples were diluted to a common concentration of 6 μg/uL. Plasma samples were diluted 1:3. Plasma and brain homogenate samples were run in singlet on a 96-well plate alongside standard curve and quality control calibration samples. Significance was assessed using Šidák’s posthoc after 1-way ANOVA.

### Proteomics

Proteins were analyzed from a 2 mm coronal section of the left hemisphere posterior to the olfactory bulb as previously described [28]. Proteomics analyses were performed at the Vincent Coates Foundation Mass Spectrometry Laboratory, Stanford University Mass Spectrometry (SUMS-RRID:SCR_017801). Lysis buffer (5% SDS, 50 mM TEAB, and 1X Protease and Phosphatase Inhibitors) was added to tissue samples, and they were homogenized by bead mill. The resulting lysate was cleared and transferred for filter supported digestion. Proteins were reduced with 10 mM DTT at 550 °C for 30 min, followed by alkylation with 30 mM acrylamide for 30 min at room temperature. 0.5 μg of Trypsin/LysC protease (Promega) was added to each sample for digestion at 37 °C overnight. After digestion, the reaction was quenched using 1% formic acid, and peptides were eluted and dried. Peptide quantification was performed with the Pierce Quantitative Fluorometric Peptide Assay kit (Thermo Fisher Scientific). The peptide mixture was dried by speed vac before dissolution in reconstitution buffer (2% acetonitrile with 0.1% formic acid). 1 μg was used for subsequent LC–MS/MS analysis. The mass spectrometry experiment was performed using an Orbitrap Eclipse Tribrid mass spectrometer RRID:022212 (Thermo Scientific, San Jose, CA, USA) with liquid chromatography using an Acquity M-Class UPLC (Waters Corporation, Milford, MA, USA). A flow rate of 300 nL/min was used, where mobile phase A was 0.2% formic acid in water and mobile phase B was 0.2% formic acid in acetonitrile. Analytical columns were prepared in-house with an I.D. of 100 microns pulled to a nanospray emitter using a P2000 laser puller (Sutter Instrument, Novato, CA, USA). The column was packed using C18 reprosil Pur 1.8 micron stationary phase (Dr. Maisch) to a length of ~ 25 cm. Peptides were directly injected onto the analytical column using a gradient (2–45% B, followed by a high-B wash) of 80 min. The mass spectrometer was operated in a data-dependent fashion using CID fragmentation for MS/MS spectra generation. For data analysis, the RAW data files were processed using Byonic v4.1.5 (Protein Metrics, Cupertino, CA, USA) to identify peptides and infer proteins. Proteolysis with Trypsin/LysC was assumed to be semi-specific, allowing for N-ragged cleavage with up to 2 missed cleavage sites. Precursor mass accuracies were held within 12 ppm and 0.4 Da for MS/MS fragments. Cysteine modified with propionamide was set as fixed modifications in the search. Proteins were held to a false discovery rate of 1%, using the standard reverse-decoy technique [53]. Significant protein-level changes were assessed using a t-test.

### Western blot

Flash-frozen, 2 mm coronal partial section containing hippocampus and cortex from the left hemisphere was homogenized in 10 ul/mg of T-PER (Tissue Protein Extraction Reagent; Thermo Scientific, Cat: 78,510, Waltham, MA, USA) with Halt Protease Inhibitor Cocktail (Thermo Scientific, Cat: 78,429, Waltham, MA, USA) and Phosphatase Inhibitor Cocktails (Abcam, Cat: ab201112, ab201113, ab201114, Cambridge, UK) on ice by sonication using Ultrasonic Probe Homogenizer (Omni International, Kennesaw, GA, USA). The homogenate was centrifuged at 12,000 rpm for 10 min at 4 °C. The protein concentration was determined using the Pierce BCA (bicinchoninic acid) protein assay kit (Pierce, Cat: 23,227 Rockford, IL, USA). Samples were prepared with Novex Bolt lithium dodecyl sulfate sample buffer and Novex Bolt sample reducing agent (Invitrogen, Cat: B0007, B0009). For some antibodies, as noted below, samples were then boiled at 95 °C for 5 min. Samples were loaded 20 ug/well in 10% or 4–12%, Bis–Tris, 1.0 mm, Mini Protein Gel (Invitrogen, Cat: NW00107BOX, NW04122BOX, Waltham, MA, USA). The protein was transferred to a polyvinylidene difluoride membrane (Abcam, Cat: ab133411, Cambridge, UK) and incubated in Intercept (TBS) Blocking Buffer (Li-cor, Cat: 927-60,001, Lincoln, NE) for 1 h at room temperature. The membranes were incubated at 4 °C overnight on a shaker with anti-Akt (1:2000, Cell Signaling, Cat: 2920, Danvers, MA, USA), anti-Atg5 (1:1000, Cell Signaling, Cat: 12,994, Danvers, MA, USA), anti-DAPK1 (1:1000, Cell Signaling, Cat: 3008, Danvers, MA, USA), anti-phospho-DAPK Ser308 (1:1000, Sigma-Aldrich, Cat: D4941, Burlington, MA, USA), anti-GluA1 (1:1000, Cell Signaling, Cat: 13,185, Danvers, MA, USA), anti-LC3B (1:500, Novus Biologicals, Cat: NB100-2220, Littleton, CO, USA), anti-NR2B (1:1000, Invitrogen, Cat: MA1-2014, Waltham, MA, USA), anti-PSD95 (1:1000, Cell Signaling, Cat: 2507, Danvers, MA, USA), anti-synapsin-1 (1:1000, Cell Signaling, Cat: 5297, Danvers, MA, USA), anti-phospho-synapsin-1 Ser62, Ser67 (1:1000, Millipore, Cat: AB9848, Burlington, MA, USA), anti-synaptophysin (1:1000, Millipore, Cat: MAB329-C, Burlington, MA, USA), and anti-tubulin (1:1000, Sigma-Aldrich, Cat: T5168, Burlington, MA, USA) primary antibodies. Samples were boiled, as described above, except for those used with anti-Akt, anti-Atg5, anti-DAPK1, and anti-NR2B. The following day, membranes were washed (4 × 10 min) with 0.01% Tween-20 in 1 × TBS and incubated for 1 h at room temperature on a shaker with IRDye IgG Secondary Antibody (1:10,000, goat anti-mouse Cat: 926–68,070, goat anti-rabbit Cat: 926–32,211, goat anti-mouse Cat: 926–32,210, goat anti-rabbit Cat: 926–68,071, Li-cor, Lincoln, NE, USA). Following secondary antibody incubation, membranes were washed (4 × 10 min) with 0.01% Tween-20 in 1 × TBS. Membranes were then scanned with the Sapphire Biomolecular Imager (Azure Biosystems, Dublin, CA, USA) in the appropriate wavelengths. Azurespot Version 2.0 (Azure Biosystems, Dublin, CA, USA) was used for densitometry analysis of target protein levels and normalized to internal level of tubulin or the non-phosphorylated protein for each sample as a control. In order to stain membranes with multiple primary antibodies, membranes were stripped by incubating in 1X Restore Fluorescent Western Blot Stripping Buffer (Thermo Scientific, Cat: 62,300, Waltham, MA, USA) for 20 min at room temperature on a shaker. Membranes were washed (4 × 5 min) with 0.01% Tween-20 in 1 × TBS and incubated in Intercept (TBS) Blocking Buffer for 1 h at room temperature. Membranes were then incubated in respective primary antibodies overnight as described above.

### Analysis of clinical records

We designed an observational study to assess the effects of a pediatric exposure to ADRB2 agonists on schizophrenia-associated in-patient visits following a young-adult diagnosis of schizophrenia. The Optum Clinformatics™ Data Mart Database (OptumInsight, Eden Prairie, MN) is a de-identified database from a large national insurance provider. The dataset contains over 88 million patients largely under the age of 65 and is frequently used for observational studies. We used a version of Optum standardized to OHDSI’s Observational Medical Outcomes Partnership (OMOP) common data model (CDM) version 5 (https://github.com/OHDSI/CommonDataModel). The OMOP CDM used standard vocabulary concepts mapped to international coding systems into a consolidated data resource.

We implemented the analysis using the CohortMethod package [38] in R to fit a Cox Regression model for assessing differences between target and comparator patient populations. We designed the study following OHDSI guidelines and generated parameterized code to assess data from the Optum data set in CDM v 5.0 and make this code available in the *EHR_code* folder in the GitHub. The full, anonymized analysis pipeline is contained in EHR_code/count_schizophrenia_drugs_patiens_beta2agonists_PrimaryDiagnosis_anonymized.R. This analysis consisted of multiple selection steps and to increase transparency we have highlighted which script completes each step:

- We selected all child drug concepts relative to the “Selective beta-2-adrenoreceptor agonists“ ATC drug concept, and this included 540 individual drug concepts (e.g. “Albuterol 4 MG Oral Tablet [Nu-Salbutamol]” or “Terbutaline 5 MG Oral Tablet [Bricanyl SA] Box of 60 by Astrazeneca”); full concept list in Supplementary File 9, *EHR_code/beta2agon_pediatric_exposure_noPriorObsReq.sql*.
- We defined all young adult patients (age > 18) with a schizophrenia diagnosis using *EHR_code/Schizophrenia_young_adult_diagnosis_noPriorObsReq_v2.sql*.
- We defined our target cohort as patients with the pediatric exposure and the young adult diagnosis (*EHR_code/count_shared_patients.sql*).
- We defined our comparator cohort as young adult patients without the pediatric exposure (*EHR_code/remove_doubles_from_single_cohort.sql*).
- We required in-patient visits to be associated with a diagnosis of schizophrenia on the date of the visit or up to 3 days post visit to accommodate diagnoses logged for billing purposes (*EHR_code/inpatient_pysch_visit_new_inclusion_rule.sql*).
- We fit a large-scale propensity model to control for confounding and the model AUC was 0.87 suggesting that covariates were not sufficiently predictive of treatment assignment. We used inverse propensity weighting (IPW) and patient matching to compare target and comparator outcomes. Patient matching generates a subset of target-comparator patient comparison based on covariate similarity, but eliminates patients when a reasonable match is not available. IPW instead uses all patients but weights their contribution to hazard ratio estimation based on the representation of their covariate profile in the study population. Because we had relatively few patients, we considered both methods in this exploratory analysis. (*EHR_code/ count_schizophrenia_drugs_patiens_beta2agonists_anonymized.R*).

### Supplementary Information

Below is the link to the electronic supplementary material.

Supplementary file1 (XLSX 3946 KB)

Supplementary file2 (XLSX 40 KB)

Supplementary file3 (XLSX 67 KB)

Supplementary file4 (XLSX 21 KB)

Supplementary file5 (XLSX 75 KB)

Supplementary file6 (XLSX 16 KB)

Supplementary file7 (XLSX 28 KB)

Supplementary file8 (XLSX 3466 KB)

Supplementary file9 (XLSX 60 KB)

Supplementary file10 (PDF 1597 KB)

## Data Availability

To increase rigor and transparency, we have made code and data (except for electronic health record data) available through GitHub: https://github.com/jenwilson521/phagocytosis_beta2Agonists. Throughout the methods, we have included reference to scripts and data files that can be found in the GitHub repository.

## Code availability

To increase rigor and transparency, we have made code and data (except for electronic health record data) available through GitHub: https://github.com/jenwilson521/phagocytosis_beta2Agonists. Throughout the methods, we have included reference to scripts and data files that can be found in the GitHub repository.
