# Supplementary material for: Beta-2 adrenergic receptor agonism alters astrocyte phagocytic activity and has potential applications to psychiatric disease
Source: Discov Ment Health. 2023 Nov 30;3(1):27. doi: 10.1007/s44192-023-00050-5 (PMC10689618; doi:10.1007/s44192-023-00050-5)
Supplement: Supplementary file 10 — Supplementary file10 (PDF 1597 KB) [file 44192_2023_50_MOESM10_ESM.pdf]

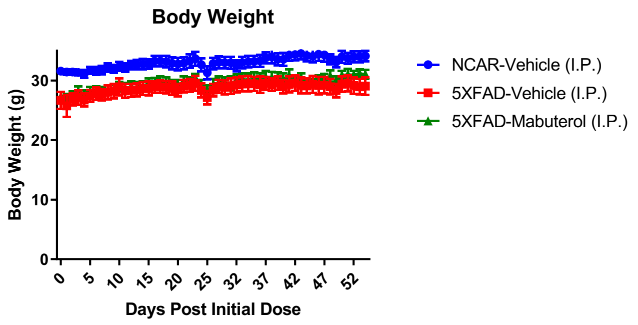

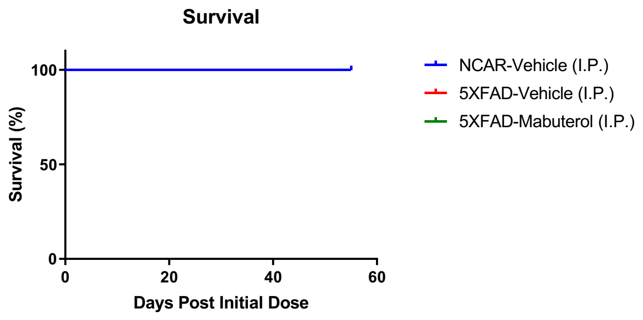

Figure S1. Mouse body weight and survival during *in vivo* study. Mice maintained body weight, and all mice survived through the study. Vehicle-treated non-carriers (NCAR-Veh), vehicle-treated 5XFAD (Hemi-Veh), and mabuterol-treated 5XFAD (Hemi-Mabuterol) are represented by blue, red, and green data curves.

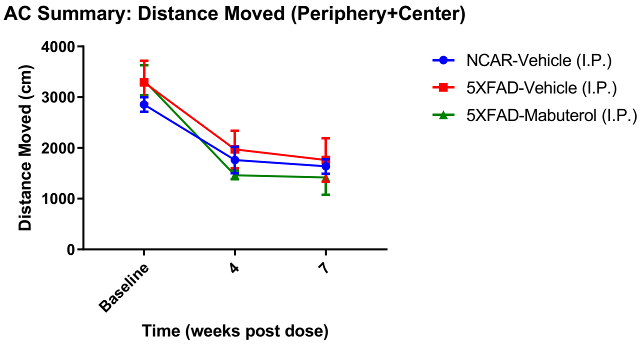

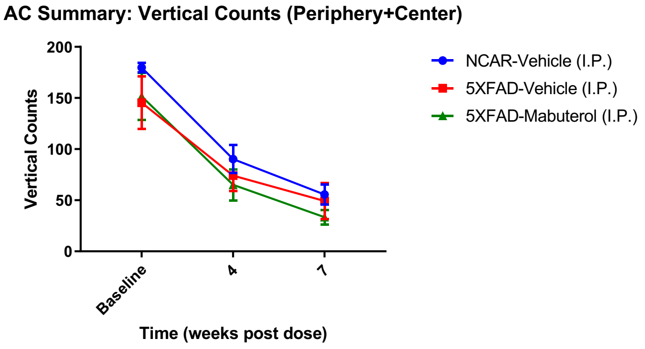

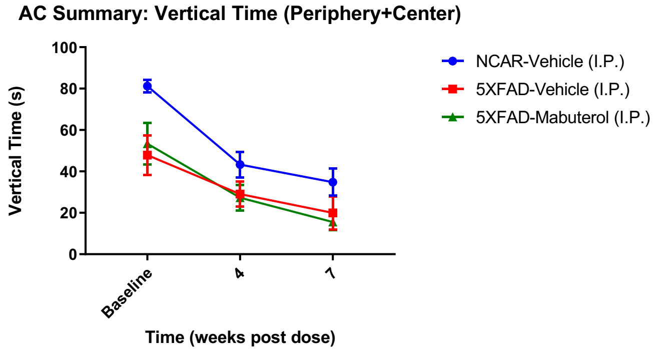

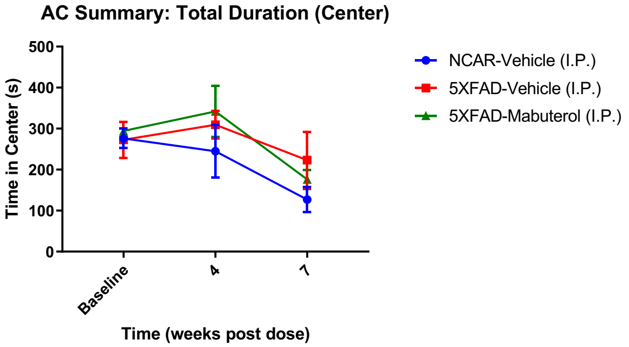

Figure S2. Activity Chamber showed no significant changes in spontaneous activity over the course of the study. We measured the distance moved (upper left), vertical counts (upper right), vertical time (lower left), and total duration spent in the center of the arena (lower right) over the course of the study and observed no statistically significant differences. Vehicle-treated non-carriers (NCAR-Veh), vehicle-treated 5XFAD (Hemi-Veh), and mabuterol-treated 5XFAD (Hemi-Mabuterol) are represented by blue, red, and green data curves.

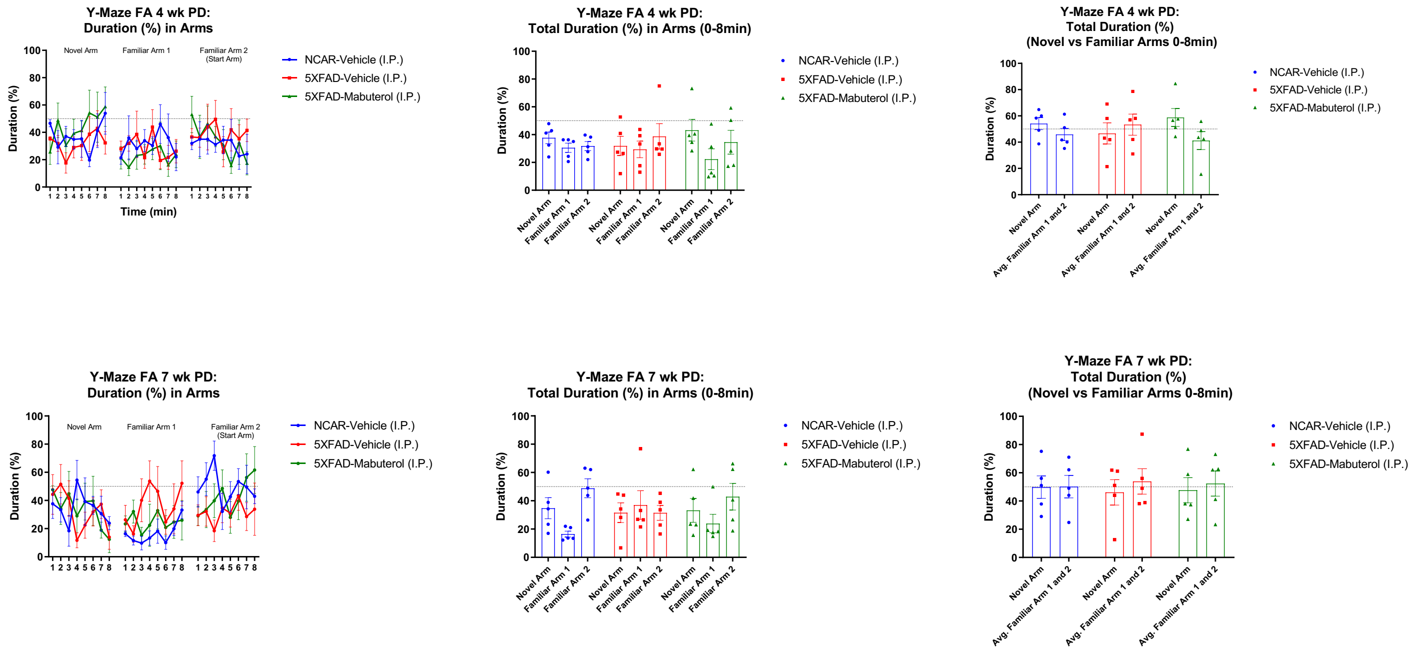

Figure S3. Y-maze: Forced Alternation showed no changes in spatial reference memory across treatment groups. We examined the total duration spent in the novel and familiar arms at 4 weeks (top) and 7 weeks (bottom) post-dosing. We analyzed this information based on the duration in each arm (left) and based on the duration in the novel arm compared to the average of the duration in the familiar arms (right). Vehicle-treated non-carriers (NCAR-Veh), vehicle-treated 5XFAD (Hemi-Veh), and mabuterol-treated 5XFAD (Hemi-Mabuterol) are represented by blue, red, and green data curves.

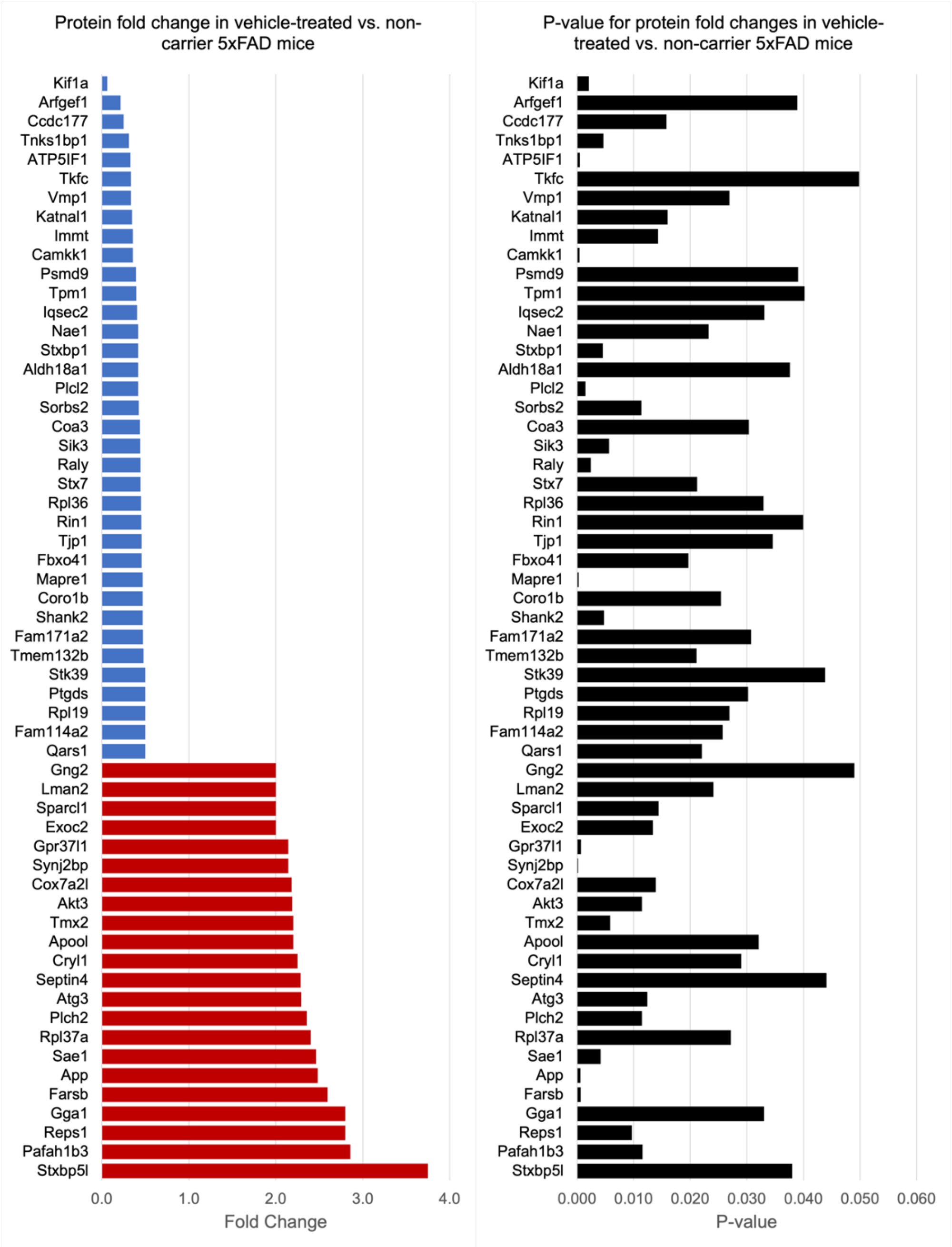

Figure S 4. Protein expression level changes detected by proteomics in 5XFAD mice compared to controls. Protein expression changes and their significance were assess for vehicle-treated 5XFAD (Hemi-Veh) compared to vehicle-treated non-carriers (NCAR-Veh) (**A**,**B**). Blue and red bars indicate down- and up- regulated proteins respectively.

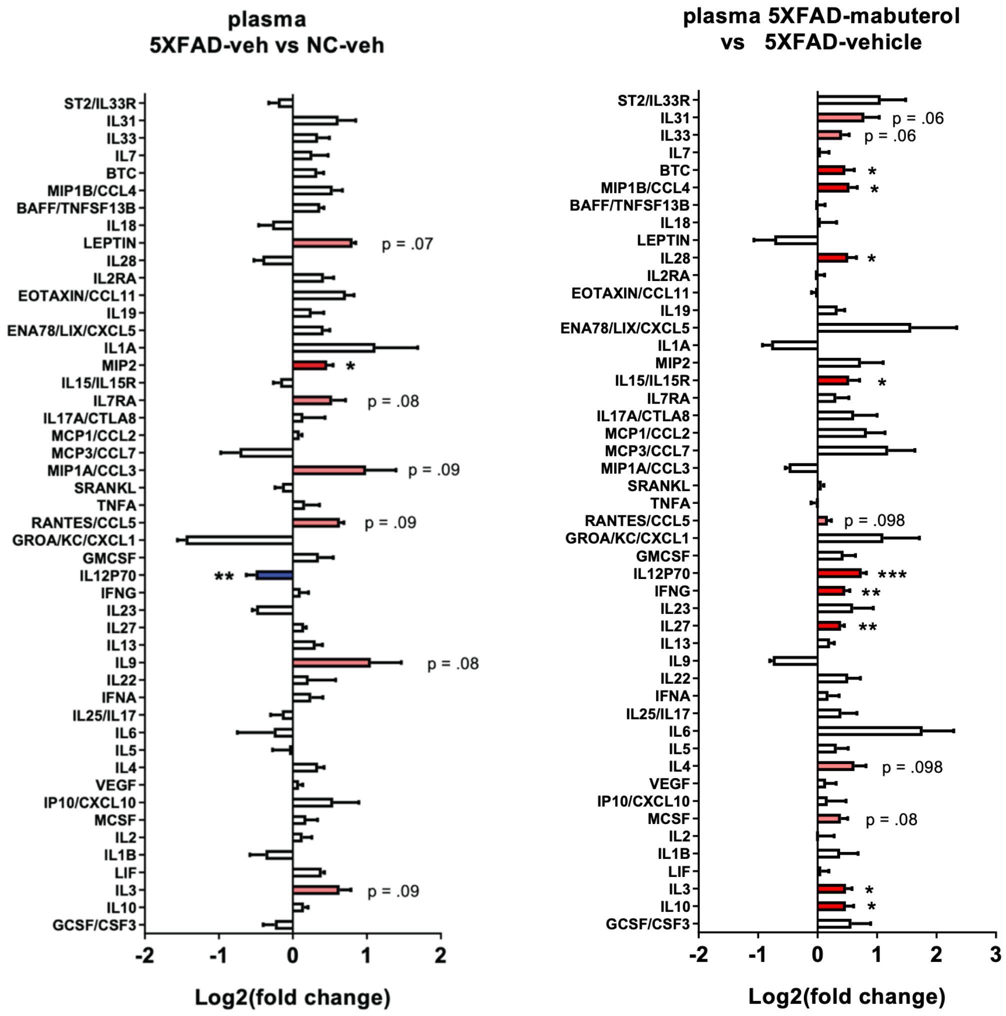

Figure S 5. Inflammatory cytokine changes in plasma detected by Luminex 48-plex (Affymetrix) mouse cytokine assay in 5XFAD mice compared to controls. We measured changes in cytokine levels comparing vehicle-treated 5XFAD (5XFAD-Veh) to vehicle-treated non-carriers (NCAR-Veh) (left) or mabuterol-treated 5XFAD (5XFAD-mabuterol) to vehicle-treated (5XFAD-vehicle) (right).

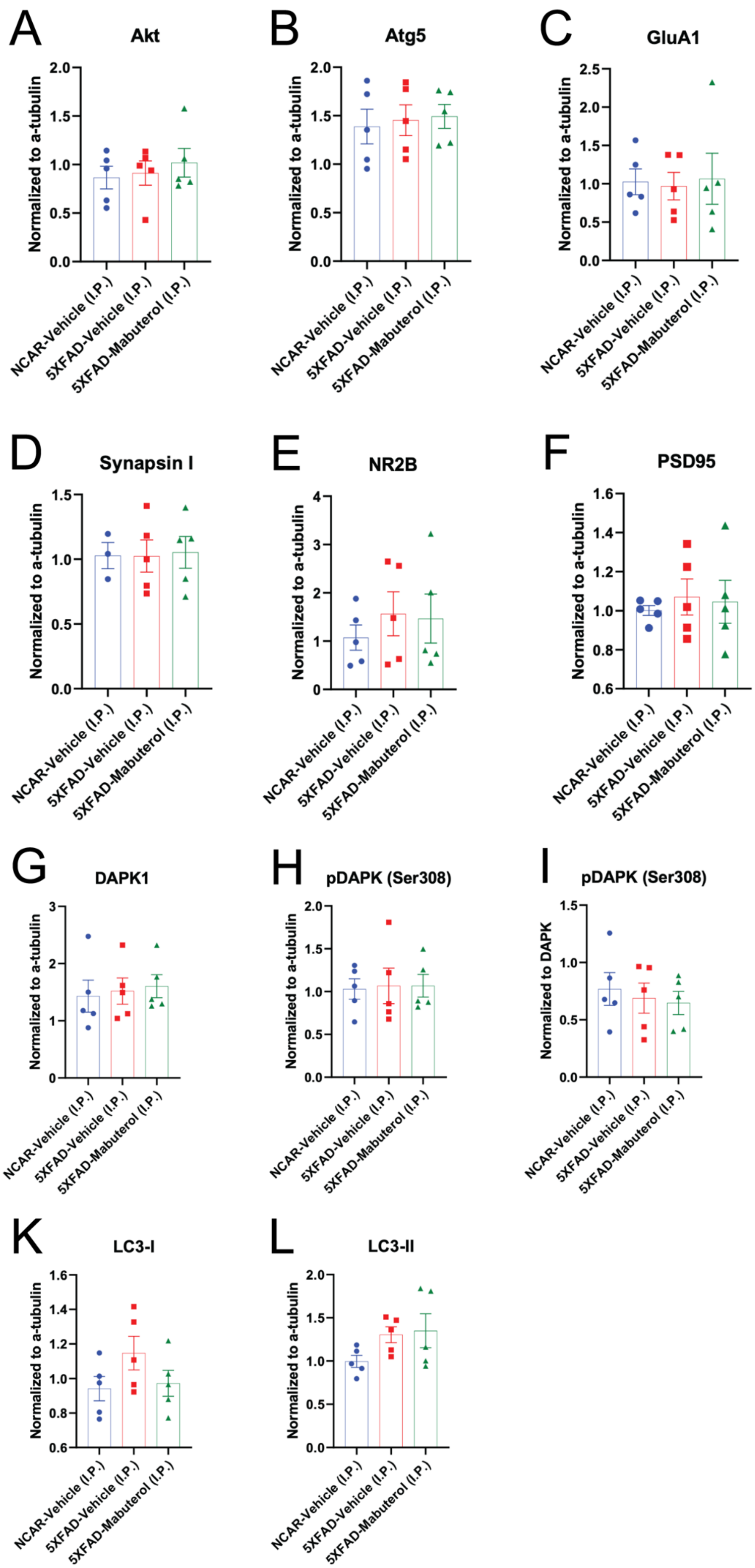

A
Akt
Normalized to a-tubulin
0.0
0.5
1.0
1.5
2.0
NCAR-Vehicle (I.P.)
5XFAD-Vehicle (I.P.)
5XFAD-Mabuterol (I.P.)
B
Atg5
Normalized to a-tubulin
0.0
0.5
1.0
1.5
2.0
NCAR-Vehicle (I.P.)
5XFAD-Vehicle (I.P.)
5XFAD-Mabuterol (I.P.)
C
GluA1
Normalized to a-tubulin
0.0
0.5
1.0
1.5
2.0
2.5
NCAR-Vehicle (I.P.)
5XFAD-Vehicle (I.P.)
5XFAD-Mabuterol (I.P.)
D
Synapsin I
Normalized to a-tubulin
0.0
0.5
1.0
1.5
NCAR-Vehicle (I.P.)
5XFAD-Vehicle (I.P.)
5XFAD-Mabuterol (I.P.)
E
NR2B
Normalized to a-tubulin
0
1
2
3
4
NCAR-Vehicle (I.P.)
5XFAD-Vehicle (I.P.)
5XFAD-Mabuterol (I.P.)
F
PSD95
Normalized to a-tubulin
0.6
0.8
1.0
1.2
1.4
1.6
NCAR-Vehicle (I.P.)
5XFAD-Vehicle (I.P.)
5XFAD-Mabuterol (I.P.)
G
DAPK1
Normalized to a-tubulin
0
1
2
3
NCAR-Vehicle (I.P.)
5XFAD-Vehicle (I.P.)
5XFAD-Mabuterol (I.P.)
H
pDAPK (Ser308)
Normalized to a-tubulin
0.0
0.5
1.0
1.5
2.0
NCAR-Vehicle (I.P.)
5XFAD-Vehicle (I.P.)
5XFAD-Mabuterol (I.P.)
I
pDAPK (Ser308)
Normalized to DAPK
0.0
0.5
1.0
1.5
NCAR-Vehicle (I.P.)
5XFAD-Vehicle (I.P.)
5XFAD-Mabuterol (I.P.)
K
LC3-I
Normalized to a-tubulin
0.6
0.8
1.0
1.2
1.4
1.6
NCAR-Vehicle (I.P.)
5XFAD-Vehicle (I.P.)
5XFAD-Mabuterol (I.P.)
L
LC3-II
Normalized to a-tubulin
0.0
0.5
1.0
1.5
2.0
NCAR-Vehicle (I.P.)
5XFAD-Vehicle (I.P.)
5XFAD-Mabuterol (I.P.)

Figure S6. Targeted Western blot analysis did not uncover any protein level changes in a handful of selected proteins. We analyzed protein level changes for Akt (**A**), Atg5 (**B**), GluA1 (**C**), synapsin I (**D**), NR2B (**E**), PSD95 (combined bands: **F**), DAPK1 (**G**), phospho-DAPK Ser308 (“pDAPK”) (normalized to alpha-tubulin: **H**, normalized to DAPK1: **I**), LC3-I (**K**), and LC3-II (**L**). Vehicle-treated non-carriers (NCAR-Veh), vehicle-treated 5XFAD (Hemi-Veh), and mabuterol-treated 5XFAD (Hemi-Mabuterol) are shown in blue, red, and green respectively.

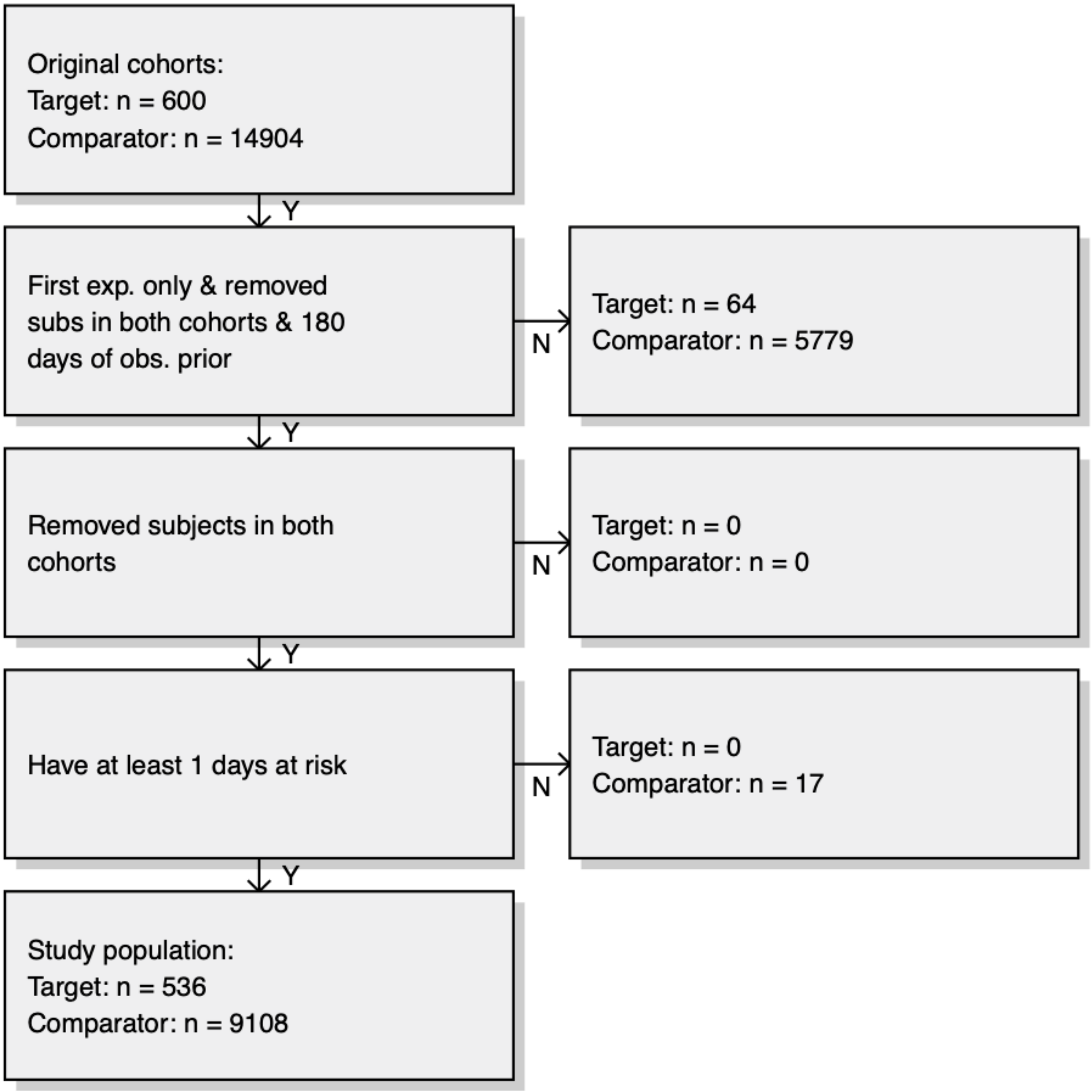

Figure S7. Observational study attrition diagram.

		Effect	Phagocytosis of synaptosomes (% of control)				
**Adrenoceptor Agonists**			**1.39 μM**	**2.78 μM**	**5.56 μM**	**11.11 μM**	**22.22 μM**
Moxonidine hydrochloride	*Alpha 2a Adrenoceptor Agonist*	↑	*118*	*127.8*	*128.5*	*130.6*	*167.2*
Salmeterol xinafoate	*Beta2 Adrenoceptor Agonist*	↓	*92*	*82.7*	*61.5*	*72.5*	*43.1*
**Sulfonylureas**							
Tolazamide		↑	*93.7*	*111.1*	*104.8*	*136.3*	*158.8*
Glibenclamide		↑	*92.6*	*134.1*	*120.2*	*104*	*138.7*
**Androgen/Estrogen**							
Triptophenolide	*Anti Androgen*	↓	*69.8*	*54.5*	*57.1*	*32.1*	*21.9*
Estradiol benzoate		↓	*4.2*	*4.2*	*33.6*	*5.5*	*69.9*
**Inflammatory**							
Prednisolone sodium phosphate		↓	*76.3*	*59.1*	*36.5*	*14.9*	*8.5*
Gitoxigenin diacetate		↓	*71.9*	*52.7*	*18.2*	*12.2*	*47.2*
**Glutamate receptor agonists**							
Kynurenic acid		↓	*107.2*	*90.3*	*67.5*	*76*	*54.6*
(±)-2-Amino-4-phosphonobutyric acid		↑	*98.6*	*101.3*	*120.2*	*132.4*	*131.5*
Acamprosate calcium		↓	*86.5*	*35.5*	*28*	*6.6*	*5.9*
**Other**							
Fluphenazine dihydrochloride		↓	*103.6*	*93.6*	*84.5*	*49*	*47.3*
Montelukast Sodium		↓	*46.4*	*2.7*	*24.9*	*28.5*	*0*

Table S1. Selected hit compounds and percent phagocytosis of synaptosomes by human astrocytes relative to control.

Molecule Name	Activity	Toxicity	Molecule Mechanism
		LOPAC Results	
Terbutaline hemisulfate	inactive	non-toxic	beta-Adrenoceptor agonist; bronchodilator
Tulobuterol hydrochloride	inactive	non-toxic	beta-Adrenoceptor agonist related to structurally to terbutaline; bronchodilator
Isotharine mesylate	inactive	non-toxic	beta-Adrenoceptor agonist; bronchodilator
(-)-Isoproterenol hydrochloride	inactive	non-toxic	beta-Adrenoceptor agonist; increases cytosolic cAMP
Salmeterol xinafoate	Decrease phagocytosis	non-toxic	Beta-2 adrenoceptor agonist
		MS Results	
Tulobuterol hydrochloride	inactive	non-toxic	beta-Adrenoceptor agonist related to structurally to terbutaline; bronchodilator
Isotharine mesylate	decrease phagocytosis	non-toxic	beta-Adrenoceptor agonist; bronchodilator
Nylidrin hydrochloride	inactive	non-toxic	beta Adrenoceptor agonist; peripheral vasodilator
Salbutamol	inactive	non-toxic	beta2-Adrenoceptor agonist
Amiodarone hydrochloride	decrease phagocytosis	toxic	alpha and beta adrenoceptor agonist; inhibits binding of 1,4-dihydropyridine to L-type Ca2+ channels; coronary vasodilator
Fenoterol hydrobromide	inactive	non-toxic	Beta2-adrenoceptor agonist; bronchodilator

Table S2. Selected adrenoceptor agonist compounds and their effects. These data are also included in **Supplementary File 1.**

Dose	1.39 uM	2.78 uM	5.56 uM	11.11 uM	22.22 uM
Percent inhibition	92	82.7	61.5	72.5	43.1

Table S3. Salmeterol had a dose-dependent decrease in phagocytosis. These data are also included in **Supplementary File 1.**

	Before matching			After matching		
	Target	Comparator		Target	Comparator	
Characteristic	%	%	Std. diff	%	%	Std. diff
Age group						
15 - 19	35.6	12.9	0.55	34.1	31.5	0.06
20 - 24	58.8	48.3	0.21	59.9	63.1	-0.07
25 - 29	5.6	32.3	-0.72	6	4.6	0.06
30 - 34		6.5			0.8	
Gender: female	29.9	34.3	-0.09	30.5	27.7	0.06
Medical history: General						
Acute respiratory disease	39	23.6	0.34	36.9	38.7	-0.04
Attention deficit hyperactivity disorder	24.8	12.8	0.31	22.6	22.6	0
Chronic obstructive lung disease	0.7	0.9	-0.01	0.8	1.6	-0.07
Depressive disorder	54.3	45.9	0.17	53.5	48.9	0.09
Gastroesophageal reflux disease	9.9	6.7	0.12	10	10	0
Hyperlipidemia	4.9	7.5	-0.11	5	4.2	0.04
Hypertensive disorder	10.1	9.8	0.01	9.8	7.4	0.09
Obesity	8.4	7.6	0.03	8.6	7.4	0.04
Urinary tract infectious disease	8.8	8.9	0	8.4	6.8	0.06
Visual system disorder	14.9	12.3	0.08	15.4	15.2	0.01
Medical history: Cardiovascular disease						
Heart disease	12.3	8.6	0.12	12.2	11.6	0.02
Medication use						
Agents acting on the renin-angiotensin system	1.9	2.1	-0.02	1.6	0.8	0.07
Antibacterials for systemic use	54.5	39.1	0.31	54.1	49.7	0.09
Antidepressants	53.2	42.3	0.22	51.7	49.5	0.04
Antiepileptics	38.4	30.2	0.17	37.1	33.9	0.07
Antiinflammatory and antirheumatic products	18.5	14.9	0.09	18.4	15.4	0.08
Beta blocking agents	7.6	5.2	0.1	7.6	7.4	0.01
Calcium channel blockers	0.9	1	-0.01	1	0.6	0.04
Drugs for acid related disorders	10.3	7.3	0.1	10.2	8.2	0.07
Drugs for obstructive airway diseases	19.2	7.7	0.34	17.4	14.8	0.07
Opioids	13.6	10.3	0.1	13.2	13	0.01
Psycholeptics	70	62.1	0.17	68.5	66.3	0.05
Psychostimulants, agents used for adhd and nootropics	22.2	11.5	0.29	21.2	20.4	0.02

Table S4. Abbreviated covariate balance between treatment and comparator groups after matching containing features with > 5% representation and a few selected cardiovascular features. Full covariate table provided in Supplementary File 9.

Cohort Descriptor	Cohort Count
beta2 agonist exposure while age <=18 and age > 6	1,295,938
schizophrenia young adult (SYA) diagnosis while age >= 18	15,504
patients with pediatric exposure and SYA diagnosis	600
SYA diagnosis minus those with pediatric exposure	14,904
In patient visits associated with a schizophrenia diagnosis (SZD)	31,292
SYA patients with pediatric exposure and subsequent SZD inpatient visits	15
SYA patients without pediatric exposure and subsequent SZDinpatient visits	1,075

Table S5. Patient counts for analysis of effect of exposure to beta-2 agonists on schizophrenia-associated (SZD) in-patient visits in young adult patients.
